# Design, synthesis and structural studies of *meta*-xylyl linked *bis*-benzimidazolium salts: potential anticancer agents against ‘human colon cancer’

**DOI:** 10.1186/1752-153X-6-68

**Published:** 2012-07-18

**Authors:** Rosenani A Haque, Muhammad Adnan Iqbal, Mohamed B Khadeer Ahamed, AMS Abdul Majid, Zena A Abdul Hameed

**Affiliations:** 1 School of Chemical Sciences, Universiti Sains Malaysia, USM-11800, Penang, Malaysia; 2 EMAN Research and Testing Laboratory, School of Pharmacy, Universiti Sains Malaysia, USM-11800, Penang, Malaysia

## Abstract

**Background:**

Benzimidazole derivatives are structurally bioisosteres of naturally occurring nucleotides, which makes them compatible with biopolymers of living systems. This property gives benzimidazole a biological and clinical importance. In the last decade, this class of compounds has been reported to possess anti-allergic, anti-diabatic, anti-HIV, anti-hypertensive, anti-inflammatory, anti-mycobacterial, anti-oxidant, anti-protozoal, and anti-viral properties. The researchers are now interested to explore their potential as anti-cancer agents. In the present study, an effort was made to further explore this area of research. Furthermore, in order to increase the solubility and efficacy of these heterocycles, the interest is now shifted to the salts of these compounds. With this background, we planned to synthesize a series of *meta*-xylyl linked *bis*-benzimidazolium salts to assess their anti-proliferation efficacy on human colon cancer cell line (HCT 116).

**Results:**

A number of *N*-alkylbenzimidazoles were synthesized by reactions of benzimidazole with alkyl halides (*i*-PrBr, PrBr, EthBr, Pent-2-ylBr, BuBr, BenzCl, HeptBr). The subsequent treatment of the resulting *N*-alkylbenzimidazoles with 1,3-(bromomethylene)benzene afforded corresponding *bis*-benzimidazolium salts. All synthesized compounds were characterized by spectroscopic techniques (Additional file 1: NMR & FT-IR) and microanalysis. Molecular structures of selected compounds were established through single crystal x-ray diffraction studies. All the compounds were assessed for their anti-proliferation test on human colorectal cancer cell line (HCT 116). Results showed that the compounds exhibited dose dependent cytotoxicity towards the colon cancer cells with IC_50_ ranges between 0.1 to 17.6 μM. The anti-proliferation activity of all compounds was more pronounced than that of standard reference drug 5-flourouracil (IC_50_ =19.2 μM).

**Conclusions:**

All the synthesized *bis*-benzimidazolium salts showed potential anticancer activity. Out of them, some of these salts showed IC_50_ value as low as 0.1–0.2 μM. Based on the results it can be concluded that, the *bis*-benzimidazolium salts could probably be the potential source of chemotherapeutic drugs.

## Background

New drugs to fight cancer are constantly needed. Colon cancer (bowel cancer) is a cancer caused by uncontrolled cell growth in the colon, rectum, or vermiform appendix. It is the third most common cancer and the fourth most frequent cause of cancer deaths worldwide [1]. Every year, more than 945000 people develop colorectal cancer worldwide, and around 492000 patients die [1].

Benzimidazole is a heterocyclic moiety possessing wide spectrum of biological activities [2]. The biological importance of benzimidazole derivatives is due to their structural resemblance to the naturally occurring nucleotides, which allow them to interact with the biopolymers of the living system [2]. In the last decade some benzimidazole derivatives have shown some potential anticancer activities, for example compound **1** exhibited anticancer activity against MCF-7, HL-60, HT-29, and PD-3 cell lines (IC_50_ value ranging from 7.0 to 100 μM) [3]. Compound **2** has shown 51% inhibition of tumor growth in mice implanted with HT-29 human carcinoma at 400 mg/kg orally [4]. Compounds **3** and **4** have shown anticancer (A-549 IC_50_ 2.8 μM, HeLa IC_50_ 5.1 μM) and antitumor (EBV-EA) activities respectively [5,6].

**Figure 1:**
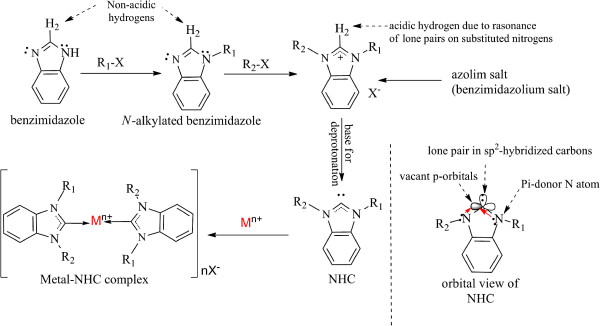
**Synthesis of free NHC from benzimidazolium salt and orbital view of a benzimidazolium-based NHC [**[10]**].**

Due to the aforementioned reasons, current study was conducted to synthesize a series of benzimidazolium salts (i.e., **9**, organic salts) unlike benzimidazole derivatives (i.e., **1–4**, organic compounds) in order to increase the solubility and efficacy of these heterocycles. It was found that these *bis*-benzimidazolium salts have shown activity against human colon cancer cell lines (HCT 116). Azolium (imidazolium, benzimidazolium, triazolium etc.) salts are used as stable precursors to synthesize *N*-heterocyclic carbenes (NHCs) and metal complexes [7-9] (see Figure 1). *N*-heterocyclic carbenes (NHCs) are types of carbenes in which a divalent carbon moiety is flanked by two π-donor nitrogen atoms (for example,**5** and **6**) [9]. For details see [8,9]

To synthesize NHCs, the more common practice is substitutions of alkyl/aryl groups at nitrogens of azoles (imidazole, benzimidazole, triazole etc.) to synthesize azolium salts in which H-2 hydrogen becomes acidic which can be removed by addition of a base (e.g., t-BuOK, NaH etc.) [11,12]. There are numerous ways to generate free NHCs and their metal complexes [9,13].

Here in this article we report some *meta*-xylyl linked *bis*-benzimidazolium salts of structure **9** with IC_50_ values 0.1–17.8 μM.

## Results and discussions

Most of the naturally occurring and synthetically available imidazolium or benzimidazolium derivatives are of the non-bridged variety. It was therefore of great interest to investigate the addition of an aryl alkyl spacer of 1,3-dimethylene benzene to such non-bridged systems to extend the application of direct electrophilic substitution of long and branched alkyl chain methodology.

### Syntheses

**Scheme 1:**
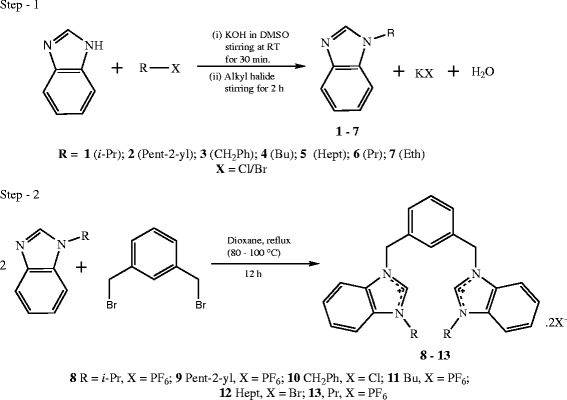
Synthesis of N-alkyl benzimidazoles (1-7) and 3,3'-(1,3-phenylene(methylene))(1-alkyl-Benzimidazolium) salts (8-13).

All the N-alkyl benzimidazoles were synthesized by the method developed by starikova et al. [14] with minor modifications. Furthermore the reaction of two equivalents of *N*-alkyl benzimidazole with 1,3-(bromomethylene)benzene in 1,4-dioxane at 80-100°C for 24 h afforded the product in 29–94% yield (Scheme 1).

We have already reported [10,11,13,15-17] the synthesis of a variety of medium- and long-chain-derivatized imidazolium and benzimidazolium salts by our simple, high-yielding, and general synthetic method. We previously employed ethyl bromide, propyl bromide, *i*-propyl bromide, butyl bromide, benzyl chloride, and heptyl bromide as the electrophiles for 1,2-(bromomethylene)benzene [17] and now show that the use of these electrophiles on 1,3-(bromomethylene)benzene is also successful. The method adopted for the synthesis of title compounds is selective for the 1-position heterocycle, and the two-step process is essentially quantitative. Indeed, upon treating alkylbenzimidazole (**1-7)** with *m-*xylyl dibromide (1,3-(bromomethylene)benzene), respective *bis-*benzimidazolium salt was observed, to give **8-13** in almost quantitative yield. Selected compounds (**9**. 2Br, **11**. 2Br &**12**. 2Br) were converted to their respective hexaflourophosphate counterparts via metathesis reaction using KPF_6_. This step is included for easier handling and better solubility. The PF_6_ salts of all the compounds are soluble in acetonitrile, DMSO, and DMF but not soluble in methanol, ethanol, and water whereas the halide salts are soluble in methanol, ethanol, DMSO, DMF and water but not soluble in acetonitrile.

### FT-IR spectra of the compounds

**Figure 2:**
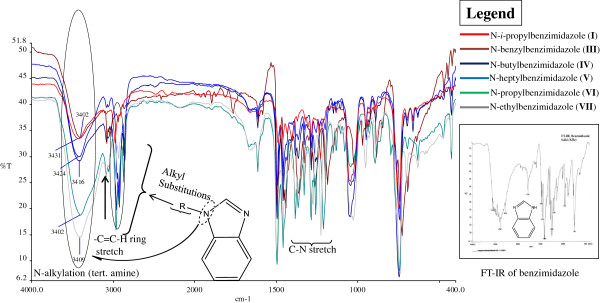
**FT-IR overlay spectrum of *N*-alkylated benzimidazoles (1-7).**

It is of much importance to study the spectral features in both near and mid IR spectra, for their strong correlation to vibrational structures of the molecules. Representative IR spectra of the compounds and the functional assignments for *N*-alkylated benzimidazoles are shown in Figure 2. For all synthesized compounds, two strong and sharp stretching vibrations (3380 – 3439 cm^−1^) appeared for the tertiary nitrogen of benzimidazolium ring in the observed spectra. The pure modes of the C-H stretching vibrational bands in both, alkyl benzimidazoles and *bis*-benzimidazolium salts appeared at around 2900 to 3000 cm^−1^. This variation in the range is due to presence of C-H (sp^3^-s) stretching of alkyl chains and methylene (N-CH_2_-Ar) group. A strong and sharp intense band observed in the range 1400 to 1450 cm^−1^ ascribed to the stretching modes of vibrations of benzimidazole ring due to the presence of -HC = N- module [18]. It may be concluded that the reduction in the intensity of this band in benzimidazolium salts is probably caused by the conjugation of C = N bond with the benzimidazole ring and due to *N*-alkylation, where alkyl group acts as electron donating entity. In benzimidazole, the modes due to the ring vibrations are characteristically strong near 1400 and 1460 cm^−1^ as are absorptions [19]. The other ring vibrations are intense bands at around 1050 and 1220 cm^−1^.

### FT-NMR spectra of the compounds

**Figure 3:**
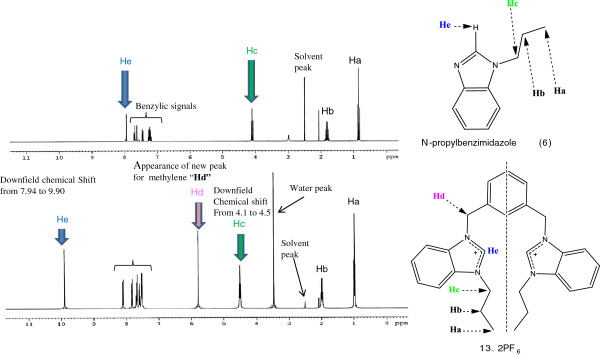
^**1**^**H NMR spectrum (*d***_***6***_**-DMSO, 400 MHz) indicating the changes in chemical shifts after successful reaction (between 6 & 13).**

FT-NMR spectra of all the compounds have been analyzed in *d*_*6*_-DMSO and *d*_*3*_-CH_3_CN over the scan range 0 to 16 δ ppm for ^1^ H NMR and 0 to 200 δ ppm for ^13^ C NMR studies. The attachment of N-alkylated benzimidazoles (**1-7**) with 1, 3-(bromomethylene) benzene, to get final products (**8-13**), was confirmed by observing changes in specific chemical shifts for N-alkylbenzimidazoles and respective salts. For example, the downfield movement of “**Hc**” signals for N-CH_2_-R from 4.1 (in N-propylbenzimidazole, **6**) to 4.5 (in its *bis*-benzimidazolium salt, **13.**2Br) and “**He**” for NCHN (acidic proton) from 7.94 to 9.90 respectively. The appearance of new signal “**Hd**” at 5.8 for N-CH_2_-Ar also supports the attachment of both the reactants (see Figure 3).

In ^13^ C NMR spectra peaks appeared between 49 to 56 ppm (see Additional file 2: Table S1). These new signals confirmed the attachment of *m*-xylene to respective *N*-substituted benzimidazoles (N-CH_2_-Ar).

In general ^1^ H NMR spectra of all the salts evidenced a sharp singlet in the range 9.88-10.27 δ ppm ascribed to the benzimidazolium ring (NCHN) acidic proton. These signals for imidazole based salts were observed in up field region, due to the absence of an electron withdrawing phenyl group [10]. Resonances of the aromatic protons of benzimidazole as well as spacer were observed in the range 7.38–8.17 δ ppm as a broad multiplet, and doublet of doublet with comparable coupling constants. The signals caused by the methylene (N-CH_2_-Ar) group, which connects xylyl unit with benzimidazolium units, display sharp singlets in the range 5.40–5.82 δ ppm. Finally, the resonance of alkyl protons appeared in the range 0.80–4.54 δ ppm with comparable coupling constants (see Additional file 2: Table S1).

Similarly, the structural assortments of the salts were further confirmed by the ^13^ C NMR spectral technique. The spectrum of all the salts evidenced a distinguished peak in the most down field range 141.81- 143.30 δ ppm ascribed to the benzimidazolium ring carbon (NCN). Resonances of aromatic carbons were found in the comparable region around 113.86–135.72 δ ppm. Also, the methylene carbon (N-C-Ar) and alkyl chain carbon resonances were observed in the chemical shift regions 49.83–55.58 and 10.82–51.70 δ ppm, respectively (See Additional file 2: Table S1).

**Figure 4:**
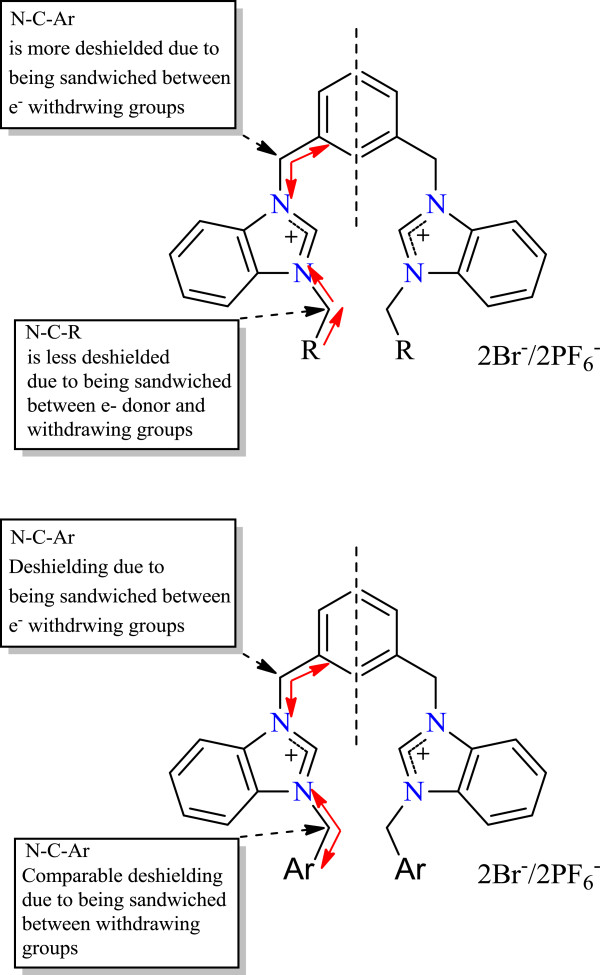
Relative deshielding of methylene groups, attached directly to benzimidazolium units in NMR spectroscopy.

In addition it is evident that N-C-Ar carbon, in all cases, is more deshielded as compared to N-C-R carbon due to being sandwiched by two electron withdrawing groups because N-C-R group is attached to electron withdrawing and donor groups (Figure 4).

### Crystallography

The molecular structures of the benzimidazolium salts Additional file 3: **10**^**.**^**2Br** and Additional file 4: **13**^**.**^**2PF**_**6**_ were determined by single crystal X-ray diffraction studies. The crystals suitable for the diffraction studies were grown by slow evaporation method. Crystal refinement data, selected bond lengths and angles of salts Additional file 3: **10**^**.**^**2Br** and Additional file 4: **13**^**.**^**2PF**_**6**_ are tabulated in Tables 1, 2, 3.

**Table 1:** Crystal data and structure refinement details for compounds 10 and 13

	**10.Br**	**13.PF**_**6**_
Formula	C_36_H_32_Br_2_N_4_	C_28_H_32_F_12_N_4_P_2_
Formula Weight	680.48	714.52
Crystal System	Triclinic	Monoclinic
Space group	P ī	Cc
a, b, c [Å]	11.0707(3), 11.3028(3), 14.8929(3)	16.2520(9), 12.0683(7), 16.2980(9)
α, β, ϒ [deg]	84.963(1), 75.975(1), 87.924(1)	90, 103.064(10), 90
V [Ang**3]	1800.80(8)	3152.2(3)
Z	2	4
D(calc)[g/cm**3]	1.255	1.506
Mu(MoKa)[/mm]	2.278	0.236
F(000)	692	1464
Crystal Size[mm]	0.56 × 0.45 × 0.29	0.58× 0.36 × 0.30
Temperature (K)	90(2)	293(2)
Radiation [Å]	MoKa 0.71073	MoKa 0.71073
*θ* Min – Max [Deg]	1.41 – 28.95	2.11 – 30.070
Dataset	−15:15;−15:15;−20 :20	−23:23;−16:16;−22:22
Tot.; Uniq. Data	33351	32572
R(int)	0.0308	0.0225
Nref;Npar	9480; 379	2023; 585
R, wR_2_, S	0.0472, 0.1376, 1.076	0.0226, 0.1293, 1.029

**Table 2:** **Selected bond lengths and angles of salt 10**^**.**^**2Br**

C6-C7	1.513(4)	C15-C16	1.515(4)	N3-C23	1.331(3)
C7-N1	1.481(3)	C16-C21	1.392(4)	C23-N4	1.332(4)
N1-C8	1.330(4)	C21-C20	1.383(4)	N4-C30	1.465(4)
N2-C8	1.339(4)	C20-C22	1.516(4)	C30-C31	1.511(4)
C15-N2	1.478(3)	C22-N3	1.464(3)	C21-H21	0.9500
C6-C7-N1	112.0(2)		C20-C22-N3	110.7(2)	
C7-N1-C8	124.5(2)		C22-N3-C23	126.1(2)	
N1-C8-N2	110.0(3)		N3-C23-N4	109.9(2)	
C8-N2-C15	125.0(2)		C23-N4-C30	125.6(2)	
N2-C15-C16	110.3(2)		N4-C30-C31	111.9(3)	

**Table 3:** **Selected bond lengths and angles of salt 13**^**.**^**2PF**_**6**_

C2-C3	1.466(7)	C11-C12	1.516(3)	N3-C25	1.317(3)
C3-N2	1.508(4)	C12-C17	1.382(2)	C25-N4	1.338(3)
N2-C4	1.325(4)	C17-C16	1.384(2)	N4-C26	1.470(3)
C4-N1	1.321(3)	C16-C18	1.509(3)	C26-C27	1.484(6)
N1-C11	1.468(3)	C18-N3	1.474(2)	P1-F1	1.506(8)
C2-C3-N2	112.4(4)		C16-C18-N3	112.19(15)	
C3-N2-C4	132.4(2)		C18-N3-C25	126.18(18)	
N2-C4-N1	111.0(2)		N3-C25-N4	110.81(18)	
C4-N1-C11	127.0(2)		C25-N4-C26	107.9(9)	
N1-C11-C12	111.95(16)		N4-C26-C27	113.9(3)	

**Figure 5:**
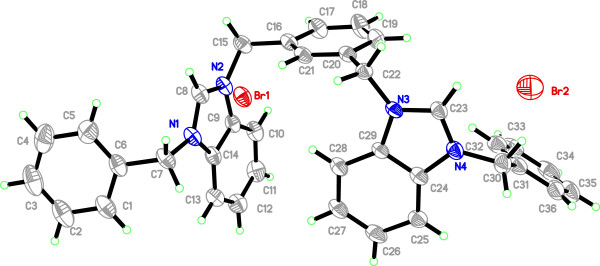
**The ORTEP picture of bis-benzimidazolium salt 10.2Br with displacement ellipsoids drawn at 50% probability.** See Figure 6A for its Packing (Triclinic).

Salt Additional file 3: **10**^**.**^**2Br** crystallizes in triclinic space group *P*-ī with one cationic *bis*-benzimidazolium core and two bromide counter ions. Crystal studies reveal the existence of salt in a zig-zag manner having benzimidazoles perpendicular to the plane of xylyl group as well as to their own planes. Similarly, the terminal phenyl groups are perpendicular to the planes of the benzimidazole rings. However, the shape of the entire molecule depends on the terminal *N-*alkyl/aryl substituent which readily offers the rigidity and finite shape. The internal imidazole ring angle (N–C–N) at the carbene center is 110.0(3)^°^ for N1-C8-N2 and 109.9(2)^°^ for N3-C23-N4, and these values are well within the range for similar benzimidazole based salts [16,20,21]. The bond angles between benzimidazole ring and xylyl were found to be N2-C9-C14 = 107.0(4) and N3-C22-C20 = 110.9(3) from opposite direction. Similarly, the bond angles of terminal phenyl ring and benzimidazole were observed in the similar manner, viz., N1-C7-C6 = 111.5(4) and N4-C24-C29 = 106.9(4). In the crystal, the bromide anions link the cations with a three-dimensional network via the intermolecular hydrogen bonding C-H….Br (2.829 and 3.040 Å) parallel to the *bc* plane. The contribution of disordered solvent molecules were removed from the diffraction data with SQUEEZE in PLATON [22,23]. A perspective view of the salt and its crystal packing are shown in Figure 5.

**Figure 6:**
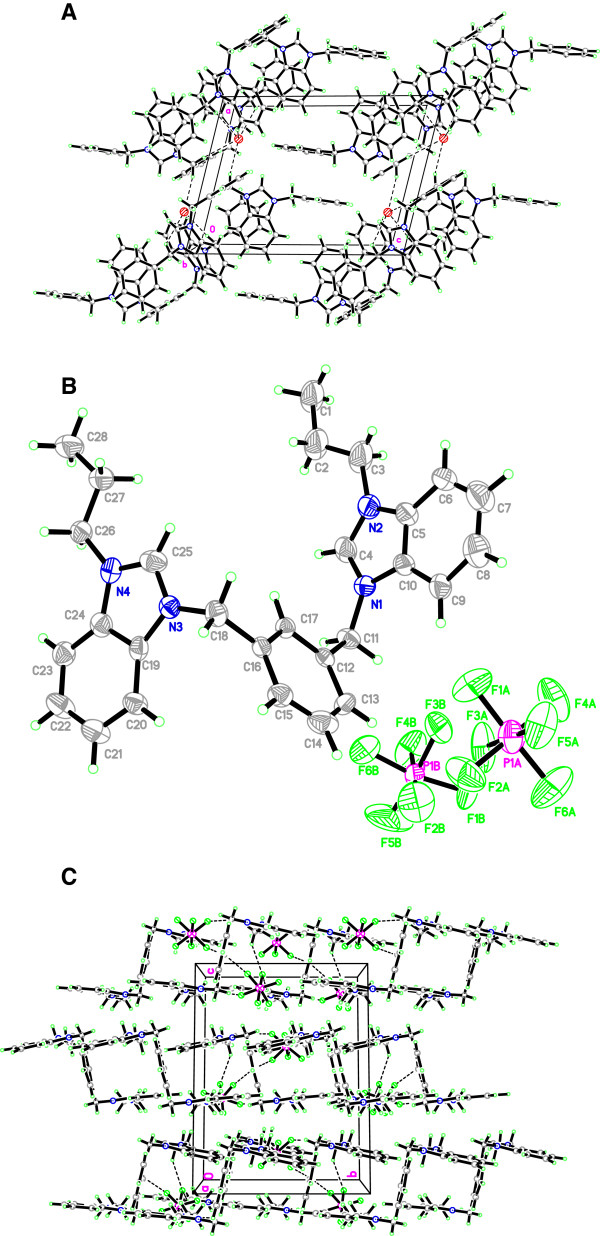
**The ORTEP picture of bis-benzimidazolium salt 13.2PF **_**6 **_**with displacement ellipsoids drawn at 50% probability and crystal packing (Monoclinic).**

On the other hand, salt Additional file 4: **13**^**.**^**2PF**_**6**_ crystallizes in monoclinic space group Cc having one cationic *bis*-benzimidazolium core and two hexafluorophosphate counter ions. The internal imidazole ring angle at the carbene center is 111.0(2)^°^ for N1-C4-N2 and 110.81(18)^°^ for N3-C25-N4, and these values are well within the range for similar benzimidazole based salts [16,20,21]. The central benzene core makes dihedral angles of 111.95(16)^°^ at N1-C11-C12 and 112.19(15)^°^ at N3-C18-C16 with the pendant benzimidazole rings on either side. The extended structure of the salt evidenced the intermolecular hydrogen bonging between cationic *bis*-benzimidazolium core and two hexafluorophosphate anions. The anions link the cations with a three-dimensional network via the C-H---F (2.638 and 2.897 Å) hydrogen bonding parallel to the *bc* plane. A perspective view of the salt and its crystal packing are shown in Figure 6.

### Anticancer activity

A wide variety of heterocyclic compounds based on imidazole, benzimidazole, pyrimidine, pyridine and purine are being intensively studied as anticancer agents against various cancer cell lines [24,25]. Furthermore, imidazole and benzimidazole-based compounds appear to be very promising candidates for anticancer treatment. As reported in recent reviews there has been a growing interest in the community of synthetic organic chemistry to examine the anticancer activities of different heterocyclic compounds with specific functional groups [26]. In this perspective, we have designed a series of *bis*-benzimidazolium salts with different counter ions.

**Figure 7:**
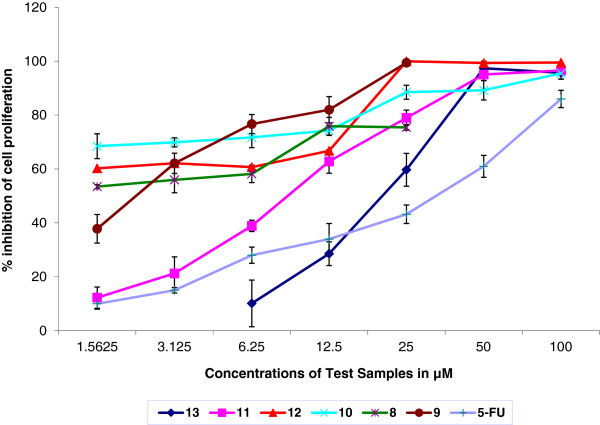
**Anti-proliferation effect of *bis-*benzimidazolium salts on HCT-116 was assessed by MTT-assay.** The figure depicts dose-dependent activity of all tested compounds. The activity of salts, 8, 9, 10 & 12 was more profound than 5-FU (values are represented as mean ± SD n=3).

**Figure 8:**
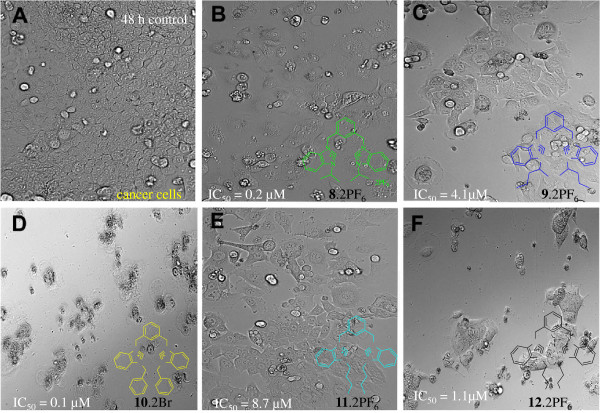
**HCT116 cell images were taken under an invented phased-contrast microscope at ×200 magnification with a digital camera at 48 hours after treatment with the samples.A**) Cells from the control group showed fully confluent growth. **B**) Treatment with **8.2**PF6 showed marked inhibition in cell proliferation with IC_50_=0.2μM. The picture revealed the autophagic characteristic in the cells as all cells treated with **8.2**PF6 showed abnormally large number of vacuoles (arrows) in cytoplasm. **C**) HCT 116 cells treated with the compound **9.2**PF6 exhibited significant cytotoxity (IC_50_=4.1μM) as the population of cells reduced drastically within the 48 hours of treatment. **D**) Photomicrograph depicts the strong cytotoxic effect of **10.2**Br (0.1μM). It can be seen clearly that the compound affected the normal morphology of most all the cells of the group which rendered the cells lose viability. **E**) Treatment with **11.2**PF6 showed the modest inhibition with IC_50_**8.7**μM. **F**) Treatment with **12.**PF6 caused sever death in HCT 116 cells as the cells appeared to be entered in apoptosis by the typhical morphological changes.

The results of the anti-proliferation test using MTT assay showed that all tested compounds exhibited a dose dependent effect. Figure 7 shows effect of different concentrations of *bis*-benzimidazolium salts (**8-13**) on human colorectal cancer cells (HCT 116) after 72 hours treatment. Anticancer efficiency of all the tested compounds is tabulated in Table 4. All the tested compounds demonstrated potent cytotoxicity against HCT 116. In particular, compounds Additional file 3: **10**^**.**^**2Br** and **8**^**.**^**2Br** were found to be the most notable examples of the series with IC_50_ values 0.1 and 0.2 μM, respectively. The results showed that the activity of these compounds was found to be more potent than the standard reference drug 5-fluorouracil (IC_50_ = 19.2 μM). Figure 8 shows the picture of cells treated with Additional file 2: **10**^**.**^**2Br** for 72 hours. Activity of these compounds is dramatically higher than the standard used. Similarly, salts **12**^**.**^**2Br** (1.1 μM) and **9**^**.**^**2PF**_**6**_ (4.1 μM) also exhibited cytotoxic effect on HCT 116, which was also stronger than the standard 5-fluorouracil (19.2 μM). In addition, salts **11**^**.**^**2PF**_**6**_ and **13**^**.**^**PF**_**6**_ also showed significant antiproliferation activity with IC_50_ values 8.7 and 17.6 μM, respectively, which is more or less equal to 5-FU.

**Table 4:** **IC**_**50 **_**Values of selected Compounds**

**Sample codes**	**IC**_**50**_**Value**
**8.**2Br	0.2 μM
**9.**2PF_6_	4.1 μM
**10.**2Br	0.1 μM
**11.**2PF_6_	8.7 μM
**12.**2Br	1.1 μM
**13.**PF_6_	17.6 μM
**5-FU**	19.2 μM

The variation in chain length will affect not only the solubility of compounds but also influences the cytotoxic property of the compounds. Thus the results showed the compounds 10.2Br (IC_50_ = 0.1 μM) with N-methylene phenyl and 12.2Br (IC_50_ = 1.1 μM) with N-heptyl substitutions revealed more potent cytotoxic activity than the compounds with smaller chain length and non-aromatic substitutions. In addition, an interesting phenomenon was noticed with the compounds having bromide anions (8.2Br, 10.2Br and 12.2Br) demonstrated significant cytotoxic activity with respect to the compounds (9.2PF_6_, 11.2PF_6_ and 13.2PF_6_) having the hexafluorophosphate anion. This data indicates that the longer alkyl chain and aromatic substitutions resulted *bis*-benzimidazolium salts more efficient compounds against HCT 116 cells.

## Conclusions

In conclusion, a series of *bis*-benzimidazolium salts was prepared in two steps. First, the preparation of *N*-alkyl benzimidazoles by the reaction of benzimidazole with appropriate alkyl halide in presence of potassium hydroxide. Second, the subsequent treatment of *N*-alkyl benzimidazole derivatives with 1,3-dibromomethylbenzene in 1,4-dioxane at refluxing temperature afforded the title compounds in good yields. Structures of selected salts were established through single crystal X-ray diffraction technique. All the synthesized salts were applied against human colon cancer cell line which showed dose-dependent cytotoxicity. Furthermore, the compounds with either bromide anion or *N*-methylene phenyl and *N*-heptyl substitutions found to be more potent cytotoxic. Thus, studies on the synthesis of respective silver complexes to observe the change in activity against human colorectal cancer cell line are in progress.

## Experimental

### Reagents and instruments

Nuclear magnetic resonance spectra were recorded on Bruker 400 MHz Ultrashield^TM^ and Bruker Avance 300 MHz spectrometers at ambient temperature. ^1^ H and ^13^ C NMR peaks are labelled as singlet (s), doublet (d), triplet (t), quartet (q), pentet (pent), sextet (sext), septet (sept) and multiplet (m), Chemical shifts were referenced with respect to solvent signals. Elemental analysis was carried out on a Perkin Elmer series II, 2400 microanalyzer. X-ray diffraction data were taken with Bruker SMART APEX2 CCD area-detector diffractometer available. The melting and boiling points were assessed by using a Stuart Scientific SMP-1 (UK) instrument. Chemicals and solvents were used as received without further purifications. All *N*-alkyl benzimidazole compounds were prepared according to the literature [14] method with slight modifications.

RPMI 1640 was purchased from ScienCell, USA. Trypsin and heat inactivated foetal bovine serum (HIFBS) were obtained from GIBCO, UK. Phosphate buffered saline (PBS), penicillin/streptomycin (PS) solution, MTT reagent and 5- fluorouracil were purchased from Sigma-Aldrich, Germany. All other chemicals used in this study were analytical grade or better.

### Syntheses

#### Synthesis of N-substituted benzimidazoles

##### Synthesis of N-isopropyl benzimidazole (**1**)

Potassium hydroxide (0.84 g, 0.015 mol) was added to a solution of benzimidazole (1.4 g, 0.01 mol) in 20 mL of DMSO, the mixture was stirred for 30 min at 18–20°C, and isopropyl bromide (1.23 g, 0.01 mol) was added dropwise under vigorous stirring and cooling with a water bath. After 2 h, the mixture was diluted with 200 ml of water and extracted with chloroform (6 × 25 ml), the combined extracts was filtered through 5 plies of whatman filter papers twice to get crystal clear solution of required product. The solvent was evaporated under reduced pressure to collect product as a thick fluid. General reaction involved in the preparation of *N*-alkyl substituted benzimidazole is shown in Scheme 1.

Appearance: Yellow oil; Yield: 86%; Boiling point: 184–188°C.

FT-IR (liquid) cm^−1^: 3324 ν (C_aliph_-N_benzimi_), 3090 and 3066 ν(C-H_arom_), 2981 and 2955 ν (C-H_aliph_), 1045 and 1229 ν (C_arom_-N_benzimi_).

^1^ H NMR (400 MHz, DMSO-*d*_*6*_) δ ppm: 1.49 (d, 6 H, 2 × CH_3_, *J* = 6.8 Hz), 4.68 (sept, 1 H, *i*-Pr-CH), 7.18 (pent, 2 H, Ar-H), 7.58 (d, 1 H, Ar-H, *J* = 8.8 Hz), 7.69 (d, 1 H, Ar-H, *J* = 7.2 Hz), 8.29 (s, 1 H, NCHN).

^13^ C NMR (100 MHz, DMSO-*d*_*6*_) δ ppm: 21.99 (2 × CH_3_), 46.95 (*i*-Pr-CH), 110.29, 119.37, 121.23, 121.99, 132.91 and 141.45 (Ar-C), 143.53 (NCHN).

##### Synthesis of N-pent-2-ylbenzimidazole (**2**)

Compound **2** was prepared according the same procedure for **1**, but instead of isopropyl bromide, 2-pentyl bromide (1.51 g, 0.01 mol) was added.

Appearance: Colorless liquid; Yield: 77%; Boiling point: 231–232°C.

FT-IR (liquid) cm^−1^: 3424 ν (C_aliph_-N_benzimi_), 3084 ν (C-H_arom_), 2966 and 2870 ν (C-H_aliph_), 1052 and 1214 ν (C_arom_-N_benzimi_).

^1^ H NMR (400 MHz, DMSO-*d*_*6*_) δ ppm: 0.75 (t, 3 H, CH_3_, *J* = 7.2 Hz), 1.46 (d, 2 H, CH_2_, *J* = 6.8 Hz), 1.72 (m, 5 H, CH_3_ & CH_2_), 4.52 (sept, 1 H, CH), 7.15 (pent, 2 H, Ar-H), 7.59 (d, 1 H, Ar-H, *J* = 9.6 Hz), 7.64 (d, 1 H, Ar-H, *J* = 8.4 Hz), 8.21 (s, 1 H, NCHN).

^13^ C NMR (100 MHz, CH_3_CN-*d*_*3*_) δ ppm: 13.72 (CH_3_), 19.73 (CH_2_), 20.98 (CH_3_), 60.58 (*i*-Pr-CH), 111.17, 117.68, 120.28, 122.17, 122.85 and 142.14 (Ar-C), 142.82 (NCHN).

##### Synthesis of N-benzyl benzimidazole (**3**)

Compound **3** was prepared according the same procedure for **1**, but instead of isopropyl bromide, benzyl chloride (1.71 g, 0.01 mol) was added. Compound appeared as white powder recrystalized by n-hexane/chloroform.

Appearance: Colorless crystals; Yield: 81%; melting point 84–86°C (Lit. mp 106–108°C [14]).

FT-IR (liquid) cm^−1^: 3402 ν (C_aliph_-N_benzimi_), 3077 and 3025 ν (C-H_arom_), 2937 ν (C-H_aliph_), 1023 and 1185 ν (C_arom_-N_benzimi_).

^1^ H NMR (400 MHz, DMSO-*d*_*6*_) δ ppm: 5.45 (s, 2 H, CH_2_), 7.16 (pent, 2 H, Ar-H), 7.28 (m, 4 H, Ar-H) 7.46 (q, 1 H, Ar-H), 7.64 (t, 1 H, Ar-H, *J* = 4.6 Hz), 8.38 (s, 1 H, NCHN).

^13^ C NMR (100 MHz, CH_3_CN-*d*_*3*_) δ ppm: 48.53 (CH_2_), 111.56 (Ar-C), 120.38, 122.50 and 123.31 (Ar-C), 128.22, 128.59 and 129.54 (Ar-C), 134.54 (Ar-C), 137.76 (Ar-C), 144.46 (Ar-C), 145.09 (NCHN).

##### Synthesis of N-butyl benzimidazole (**4**)

Compound **4** was prepared according the same procedure for **1**, but instead of isopropyl bromide, n-butyl bromide (1. 37 g, 0.01 mol) was added.

Appearance: Yellow liquid; Yield: 75.1%; Boiling point: 256–258°C.

FT-IR (liquid) cm^−1^: 3416 ν (C_aliph_-N_benzimi_), 3077 ν (C-H_arom_), 2959, 2929 and 2863 ν (C-H_aliph_), 1048 and 1165 ν (C_arom_-N_benzimi_).

^1^ H NMR (400 MHz, CD_3_CN-*d*_*3*_) δ ppm: 0.71 (t, 3 H, CH_3_, *J* = 3.8 Hz), 1.39 (sext, 2 H, CH_2_), 1.82 (pent, 2 H, CH_2_), 4.20 (t, 2 H, N-CH_2_-R, *J* = 7.2 Hz), 7.09 (pent, 2 H, Ar-H), 7.38 (d, 1 H, Ar-H, *J* = 2.8 Hz), 7.56 (d, 1 H, Ar-H, *J* = 2.8 Hz), 7.94 (s, 1 H, NCHN).

^13^ C NMR (100 MHz, CD_3_CN-*d*_*3*_) δ ppm: 13.20 (CH_3_), 19.27 (CH_2_), 31.34 (CH_2_), 43.77 (N-CH_2_-R), 109.97, 119.32, 121.18, 122.02, 133.64 and 141.57 (Ar-C), 143.59 (NCHN).

##### Synthesis of N-heptyl benzimidazole (**5**)

Compound **5** was prepared according the same procedure for **1**, but instead of isopropyl bromide, n-heptyl bromide (1.79 g, 0.01 mol) was added.

Appearance: Yellow liquid; Yield: 88.3%; Boiling point: 246–248°C.

FT-IR (liquid) cm^−1^: 3424 ν (C_aliph_-N_benzimi_), 3077 ν (C-H_arom_), 2977 and 2856 ν(C-H_aliph_), 1045 ν(C_arom_-N_benzimi_).

^1^ H NMR (400 MHz, CD_3_CN-*d*_*3*_) δ ppm: 0.85 (t, 3 H, CH_3_, *J* = 6.8 Hz), 1.22 (m, 8 H, 4 × CH_2_), 1.80 (pent, 2 H, CH_2_, *J* = 7.2 Hz), 4.13 (t, 2 H, N-CH_2_-R, *J* = 7.2 Hz), 7.18–7.26 (m, 3 H, Ar-H), 7.44 (d, 1 H, Ar-H, *J* = 9.6 Hz), 7.95 (s, 1 H, NCHN).

^13^ C NMR (100 MHz, CD_3_CN-*d*_*3*_) δ ppm: 14.08 (CH_3_), 22.76, 26.90, 29.00, 30.05 and 31.92 (alkyl chain-C), 45.12 (N-CH_2_-R), 110.59, 117.57, 120.15, 122.09, 122.93, 134.45 and 134.97 (Ar-C), 144.27 (NCHN).

##### Synthesis of N-propyl benzimidazole (**6**)

Compound **6** was prepared according the same procedure for **1**, but instead of isopropyl bromide, n-propyl bromide (1.23 g, 0.01 mol) was added.

Appearance: Colourless liquid; Yield: 87.3%; Boiling point: 236–238°C.

FT-IR (liquid) cm^−1^: 3402 ν (C_aliph_-N_benzimi_), 3084 and 3055 ν(C-H_arom_), 2966, 2929 and 2878 ν (C-H_aliph_), 1030 and 1211 ν(C_arom_-N_benzimi_).

^1^ H NMR (400 MHz, CD_3_CN-*d*_*3*_) δ ppm: 0.85 (t, 3 H, CH_3_, *J* = 7.35 Hz), 1.82 (sext, 2 H, alkyl chain-CH_2_), 4.10 (t, 2 H, N-CH_2_-R, *J* = 7.05 Hz), 7.25 (m, 2 H, Ar-H), 7.43 (d, 1 H, Ar-H, *J* = 7.2 Hz), 7.68 (d, 1 H, Ar-H, *J* = 7.2 Hz), 7.94 (s, 1 H, NCHN).

^13^ C NMR (100 MHz, CD_3_CN-*d*_*3*_) δ ppm: 11.05 (CH_3_), 23.34 (CH_2_), 46.75 (N-CH_2_-R), 110.46, 120.22, 122.03, 122.90 and 134.53 (Ar-C), 143.84 (NCHN), 144.2 (Ar-C).

##### Synthesis of N-ethyl benzimidazole (**7**)

Compound **7** was prepared according the same procedure for **1**, but instead of isopropyl bromide, ethyl bromide (1.23 g, 0.01 mol) was added.

Appearance: Colourless thick liquid; Yield 91%; Boiling Point: 184–186°C.

FT-IR (liquid) cm^−1^: 3409 (C_aliph_-N_benzimi_), 3084 (C-H_arom_), 2981, 2929 (C-H_aliph_), 1037 cm-1 (C_arom_-N_benzimi_).

1 H NMR (300 MHz, CD3CN- *d*_*3*_): δ = 1.34 (t, 3 H, CH_3_, *J* = 7.2 Hz), 4.19 (q, 2 H, CH_2_, *J* = 7.35 Hz), 7.18 (pent, 2 H, Ar-H), 7.47 (d, 1 H, Ar-H, *J* = 9.9 Hz), 7.65 (d, 1 H, Ar-H, *J* = 9.9 Hz), 8.24 (s, 1 H, NCHN).

13 C NMR (75 MHz, DMSO-*d*_*6*_): δ = 14.59 (CH_3_), 38.79 (N-CH_2_-R), 109.57, 119.17, 120.97, 121.80, 134.52, 142.70 (Ar-C), 143.68 (NCHN).

### Synthesis of N-substituted *bis*-benzimidazolium salts

#### Synthesis of 3,3'-[1,3-phenylene(methylene)]bis(1-isopropyl-benzimidazolium) dibromide (**8**.2PF_6_)

*N*-isopropyl benzimidazole (**1**) (3.2 g, 0.02 mol) was added drop wise in a vigorously stirring solution of 1,3-dibromomethyl benzene (2.64 g, 0.01 mol) in 50 ml of 1,4-dioxane and refluxed for 24 h. The product settled as a sticky brownish viscous oil at the bottom of flask, than the upper layer was decanted and product was washed with fresh dioxane (3 × 5 ml). The resulting bromide salt was converted directly to its hexaflourophosphate counterpart by metathesis reaction using KPF_6_ (0.02 mol) in 50 mL of methanol. The mixture was stirred for 24 h and filtered. The white precipitates were collected and washed with distilled water (2 × 5 mL), then left to dry at ambient temperature. General reaction involved in the preparation of *N*-alkyl substituted *bis*-benzimidazolium salts is shown in Scheme 1. The bromide salt of title compound was used for anticancer study.

Appearance: White powder; Yield: 70.1%; Melting point: 165-168°C.

FT-IR (solid): 3431 (C_aliph_-N_benzimi_), 3166, 3099 (C-H_arom_), 2981, 2858, 2828 (C-H_aliph_), 1048, 1133, 1203 cm-1 (C_arom_-N_benzimi_).

^1^ H NMR (400 MHz, DMSO-*d*_*6*_): δ = 1.64 (d, 6 H, 2 × CH_3_, *J* = 6.8 Hz), 5.05 (hept, 2 H, *i*-Pr 2 × CH, *J* = 6.6 Hz), 5.75 (s, 4 H, 2 × N-CH2-Ar), 7.42-7.70 (br.m, 8 H, Ar-H), 7.78 (d, 2 H, Ar-H, *J* = 8.4 Hz), 8.12 (d, 2 H, Ar-H, *J* = 8.40), 10.03 (s, 2 H, 2 × NCHN).

^13^ C NMR (100 MHz, DMSO-*d*_*6*_): δ = 22.28 (CH_3_), 50.65 (CH_2_), 51.70 (*i*-Pr-CH), 114.58, 115.02 (Ar-C), 127.48, 127.53, 129.32, 131.57, 131.77, 135. 60 (Ar-C) and 141.80 (NCHN).

Anal. Calcd for C_28_H_32_F_12_N_4_P_2_: C, 47.07; H, 4.51; N, 7.84. Found: C, 46.92; H, 4.48; N, 7.55.

#### Synthesis of 3,3'-[1,3-phenylene(methylene)]bis(1-(pentan-2-yl)-benzimidazolium) bis(hexaflourophosphate) (**9**.2PF_6_)

Compound **9.**2PF_6_ was prepared according to the same procedure for **8.**2PF_6_, but instead of **1**, compound **2** (3.76 g, 0.02 mol) was added.

Appearance: White powder; Yield: 29.4%; Melting point: 182–184°C.

FT-IR (solid): 3431 (C_aliph_-N_benzimi_), 3151, 3092 (C-H_arom_), 2959, 2870 (C-H_aliph_), 1052, 1111, 1192 cm-1 (C_arom_-N_benzimi_).

^1^ H NMR (300 MHz, DMSO-*d*_*6*_): δ = 0.88 (t, 6 H, 2 × CH_3_, *J* = 7.5 Hz), 1.24 (m, 2 H, CH_2_), 1.63 (m, 2 H, CH_2_), 1.97 (m, 2 H, CH_2_), 2.36 (s, 10 H, CH_3_ and _CH2_), 4.96 (sept, 2 H, CH), 5.40 (d, 4 H, N-CH_2_-Ar, *J* = 11.7 Hz), 7.46–8.17 (br.m, 12 H, Ar-H), 10.16 (d, 2 H, 2 × NCHN, *J* = 13.8 Hz).

^13^ C NMR (75 MHz, DMSO-*d*_*6*_): δ = 14.31 (CH_3_), 19.42 (CH_2_), 20.66 (CH_3_), 38.20 (CH_2_), 50.70 (pent-2-yl CH), 55.58 (N-CH_2_-Ar), 114.18 (Ar-C), 127.58, 129.32, 129.50, 130.62, 131.7 (Ar-C), and 135.56, 135.75 (NCHN).

Anal. Calcd for C_32_H_32_F_12_N_4_P_2_: C, 49.87; H, 5.23; N, 7.27. Found: C, 49.34; H, 5.19; N, 7.12.

#### Synthesis of 3,3'-[1,3-phenylene(methylene)]bis(1-benzyl-benzimidazolium) dibromide (**10**.2Br)

Compound **3** (4.16 g, 0.02 mol) was added drop wise in a vigorously stirring solution of 1,3-dibromomethylene benzene (0.01 mol) in 50 ml of 1,4-dioxane and refluxed for 24 h. The product precipitated after completion of the reaction, the solution was filtered to collect the suspended material, washed with fresh 1,4-dioxane (3 × 5 mL). The beige coloured lumps were air dried for 24 h and ground to fine powder. In this case, the bromide salt is stable and hence there is no need for the conversion into PF_6_ salt.

Appearance: beige colored solid; Yield: 51.7%; Melting point: 120–123°C.

FT-IR (solid): 3409 (C_aliph_-N_benzimi_), 3121, 3025 (C-H_arom_), 2959 (C-H_aliph_), 1015, 1185 cm-1 (C_arom_-N_benzimi_).

^1^ H NMR (300 MHz, CD_3_CN-*d*_*3*_): δ = 5.74 (s, 4 H, 2 × CH_2_), 5.78 (d, 4 H, 2 × CH_2_, *J* = 4.00 Hz), 7.38-7.98 (br.m, 22 H, Ar 22 × CH), 10.18 (s, 2 H, 2 × NCHN).

^13^ C NMR (100 MHz, CD_3_CN-*d*_*3*_): δ = 51.11, 51.34 (CH_2_), 68.2 (CH_2_), 117.2 (CH), 119.5 (CH), 128.4 – 135.9 (Ar-C), 142.3 (NCHN).

Anal. Calcd for C_36_H_32_Br_2_N_4_: C, 63.54; H, 4.74; N, 8.23. Found: C, 63.15; H, 4.68; N, 8.16.

#### Synthesis of 3,3'-[1,3-phenylene(methylene)]bis(1-butyl-benzimidazolium) bis(hexaflourophosphate) (**11**.2PF_6_)

Compound **11**.2PF_6_ was prepared according to the same procedure for **8.**2PF_6_, but instead of **1**, compound **4** (3.48 g, 0.02 mol) was added.

Appearance: White solid; Yield: 46.5%; Melting point: 230–233°C.

FT-IR (solid): 3439 (C_aliph_-N_benzimi_), 3158, 3099 (C-H_arom_), 2929, 2959, 2870 (C-H_aliph_), 1052, 1111, 1203 cm-1 (C_arom_-N_benzimi_).

^1^ H NMR (400 MHz, DMSO-*d*_*6*_): δ = 0.95 (t, 6 H, 2 × CH_3_, *J* = 5.6 Hz), 1.39 (m, 4 H, 2 × CH_2_), 1.92 (m, 4 H, 2 × CH_2_), 4.50 (t, 4 H, 2 × N-CH_2_-R, *J* = 7.2 Hz), 5.75 (s, 4 H, 2 × N-CH_2_-Ar), 7.46-7.80 (br.m, 12 H, Ar-H), 9.83 (s, 2 H, 2 × NCHN).

^13^ C NMR (100 MHz, DMSO-*d*_*6*_): δ = 14.14 (CH_3_). 19.93 (CH_2_), 31.28, 47.51 (CH_2_), 50.64, 50.83 (N-CH_2_-Ar), 114.52, 114.69 (Ar-C), 127.58, 129.32, 130.71, 132.22, 135.5 (Ar-C), 143.20 (NCHN).

Anal. Calcd for C_30_H_36_F_12_N_4_P_2_: C, 48.52; H, 4.89; N, 7.55. Found: C, 48.13; H, 4.48; N, 7.34.

#### Synthesis of 3,3'-[1,3-phenylene(methylene)]bis(1-heptyl-benzimidazolium) dibromide (**12**.2Br)

Compound **12**.2Br was prepared according to the same procedure for **10**.2Br, but instead of compound **3**, compound **5** (4.32 g, 0.02 mol) was added.

Appearance: Light brown coloured powder; Yield: 93.5%; Melting point: 249–251°C.

FT-IR (solid): 3416 (C_aliph_-N_benzimi_), 3121, 3011 (C-H_arom_), 2922, 2848 (C-H_aliph_), 1008, 1214 cm-1 (C_arom_-N_benzimi_).

^1^ H NMR (300 MHz, DMSO-*d*_*6*_): δ = 0.82 (t, 6 H, 2 × CH_3_, *J* = 6.6 Hz), 1.22-1.32 (br.d, 16 H, alkyl chain 8 × CH_2_, *J* = 29.7 Hz), 1.94 (pent, 4 H, 2 × CH_2_), 4.54 (t, 4 H, 2 × N-CH_2_-R, *J* = 7.35 Hz), 5.82 (s, 4 H, 2 × N-CH_2_-Ar), 7.45-7.68 (br.m, 8 H, Ar-H), 7.92 (d, H, Ar-CH, *J* = 8.1 Hz), 7.93 (d, 2 H, Ar-H, *J* = 8.4 Hz), 10.27 (s, 2 H, 2 × NCHN).

^13^ C NMR (75 MHz, DMSO-*d*_*6*_): δ = 10.02 (CH_3_), 13.46, 18.57, 19.82, 26.50, 37.24 (alkyl chain 5 × CH_2_), 49.85 (N-CH_2_-R), 54.37 (N-CH_2_-Ar), 114.20 (Ar-C), 126.74, 128.48, 128.66, 129.77, 130.88, 130.95, 134.71, 134.90 (Ar-C), 141.40, 143.08 (NCHN).

Anal. Calcd for C_36_H_48_Br_2_N_4_: C, 62.07; H, 6.95; N, 8.04. Found: C, 61.70; H, 6.72; N, 7.97.

#### Synthesis of 3,3'-[1,3-phenylene(methylene)]bis(1-propyl-benzimidazolium) bis(hexaflourophosphate) (**13**.2PF_6_)

Compound Additional file 4: **13.2PF**_**6**_ was prepared according to the same procedure for **8.**2PF_6_, but instead of **1**, compound **6** (3.2 g, 0.02 mol) was added. For characterization see [10].

### In vitro Anticancer activity

#### Preparation of cell culture

Initially, HCT 116 cells were allowed to grow under optimal incubator conditions. Cells that had reached a confluence of 70-80% were chosen for cell plating purposes. Old medium was aspirated out of the plate. Next, cells were washed using sterile phosphate buffered saline (PBS) (pH 7.4), 2-3 times. PBS was completely discarded after washing. Following this, trypsin was added and distributed evenly onto cell surfaces. Cells were incubated at 37°C in 5% CO_2_ for 1 min. Then, the flasks containing the cells were gently tapped to aid cells segregation and observed under inverted microscope (if cells segregation is not satisfying, the cells will be incubated for another minute) Trypsin activity was inhibited by adding 5 ml of fresh complete media (10% FBS). Cells were counted and diluted to get a final concentration of 2.5 × 10^5^ cells/mL, and inoculated into wells (100 μL cells/well). Finally, plates containing the cells were incubated at 37°C with an internal atmosphere of 5% CO_2_.

#### MTT assay

Cancer cells (100 μL cells/well, 1.5 × 10^5^ cells/mL) were inoculated in wells of microtitre plate. Then the plate was incubated overnight in CO_2_ incubator in order to allow the cell for attachment. 100 μL of test substance were added into each well containing the cells. Test substance was diluted with media into the desired concentrations from the stock. The plates were incubated at 37°C with an internal atmosphere of 5% CO_2_. After 72 hours treatment period, 20 μL of MTT reagent was added into each well and incubated again for 4 hours. After this incubation period, 50 μL of MTT lysis solution (DMSO) was added into each well. The plates were further incubated for 5 min in CO_2_ incubator. Finally, plates were read at 570 and 620 nm wavelengths using a standard ELISA microplate reader. Data were recorded and analyzed for the assessment of the effects of test substance on cell viability and growth inhibition. The percentage of growth inhibition was calculated from the optical density (OD) that was obtained from MTT assay. 5-FU was used as the standard reference drug.

## Competing interests

The authors declare that they have no competing interests.

## Authors’ contributions

RAH designed and supervised the project. MAI synthesized and characterized the compounds and AMSAM, MBK, MAI & ZAH conducted the anticancer activity. All authors read and approved the final manuscript.

## Supplementary Material

Additional file 1 Labelled Spectra (NMR & FT-IR). Click here for file

Additional file 2 **Table S1.** Some characteristic signals of bis-benzimidazolium salts in NMR Spectroscopy. Click here for file

Additional file 3 CIF file of 10. 2Br. Click here for file

Additional file 4 **CIF file 13.2PF**_**6**_**.** Click here for file
