# Supplementary material for: Design, synthesis and structural studies of meta-xylyl linked bis-benzimidazolium salts: potential anticancer agents against ‘human colon cancer’
Source: Chem Cent J. 2012 Jul 18;6:68. doi: 10.1186/1752-153X-6-68 (PMC3542276; doi:10.1186/1752-153X-6-68)
Supplement: Additional file 1 — Labelled Spectra (NMR & FT-IR). [file 1752-153X-6-68-S1.pdf]

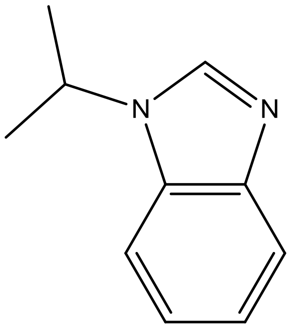

N-*i*-propyl benzimidazole (**I**)

1. FT-IR
2. ^{1}HNMR
3. ^{13}CNMR

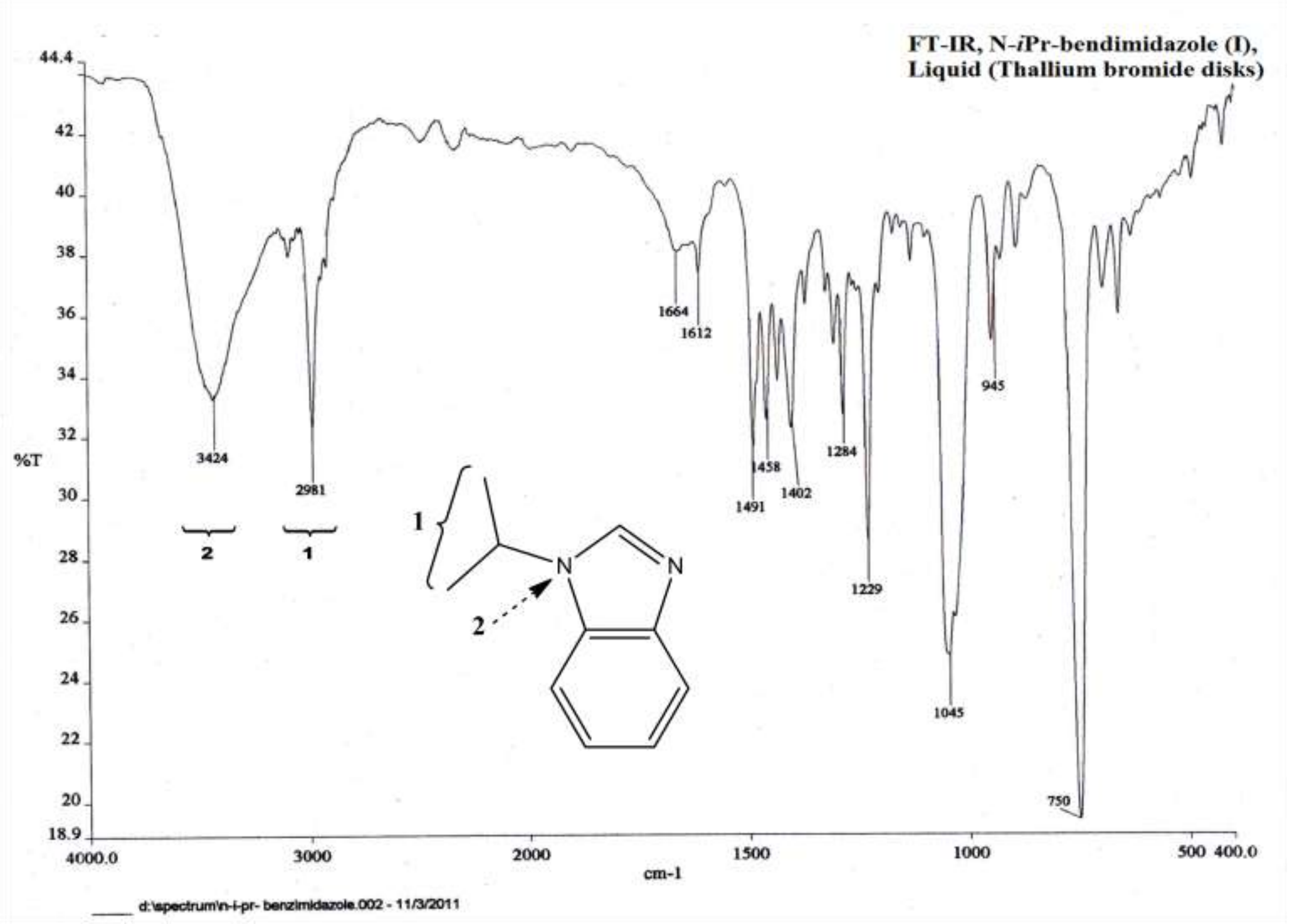
FT-IR, N-*i*Pr-bendimidazole (I),
Liquid (Thallium bromide disks)
44.4
42
40
38
36
34
32
%T
30
28
26
24
22
20
18.9
4000.0
3000
2000
1500
1000
500 400.0
cm-1
3424
2981
1664
1612
1491
1458
1402
1284
1229
1045
945
750
2
1
N
N
d:\spectrum\n-i-pr- benzimidazole.002 - 11/3/2011

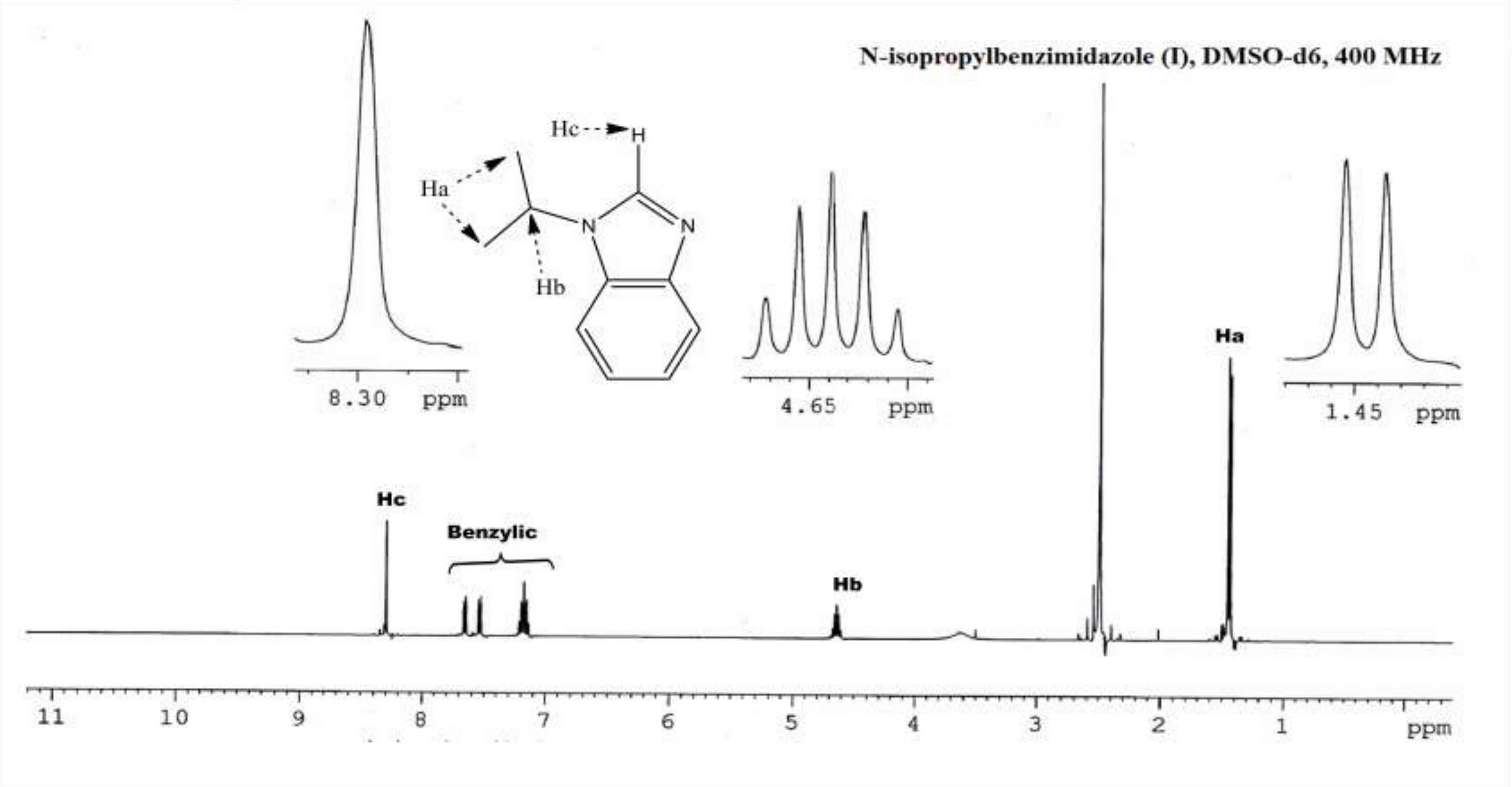
N-isopropylbenzimidazole (I), DMSO-d6, 400 MHz
Hc
H
Ha
N
N
Hb
8.30 ppm
4.65 ppm
1.45 ppm
Hc
Benzylic
Hb
Ha
11
10
9
8
7
6
5
4
3
2
1
ppm

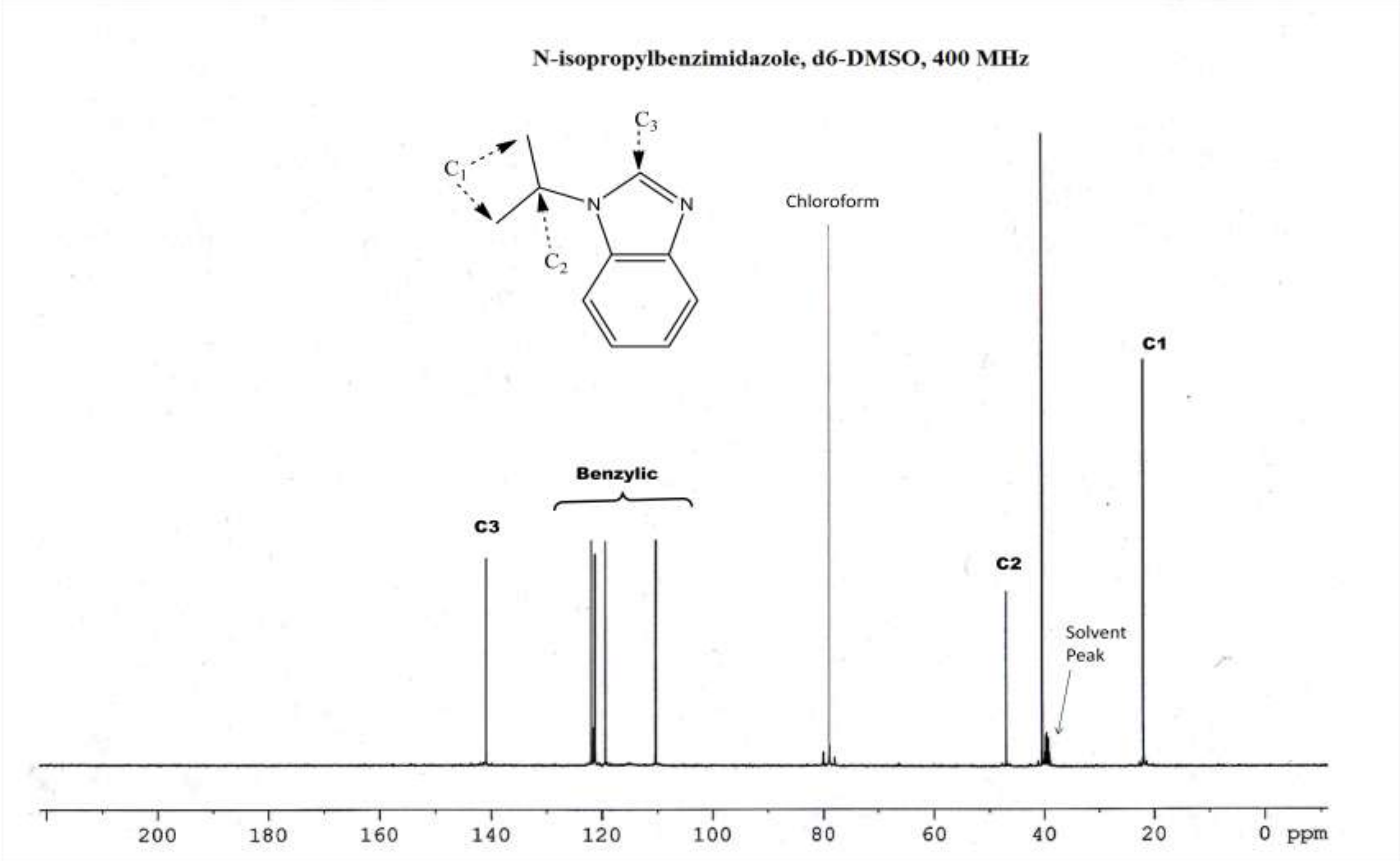
N-isopropylbenzimidazole, d6-DMSO, 400 MHz
C3
C1
N
N
C2
Chloroform
C1
Benzylic
C3
C2
Solvent
Peak
200
180
160
140
120
100
80
60
40
20
0 ppm

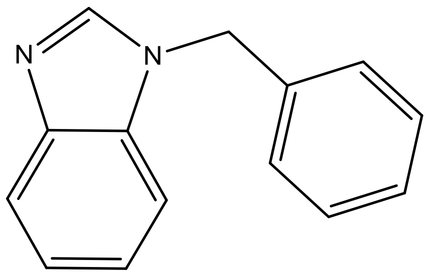

N-benzylbenzimidazole (**III**)

1. FT-IR
2. ^{1}HNMR
3. ^{13}CNMR

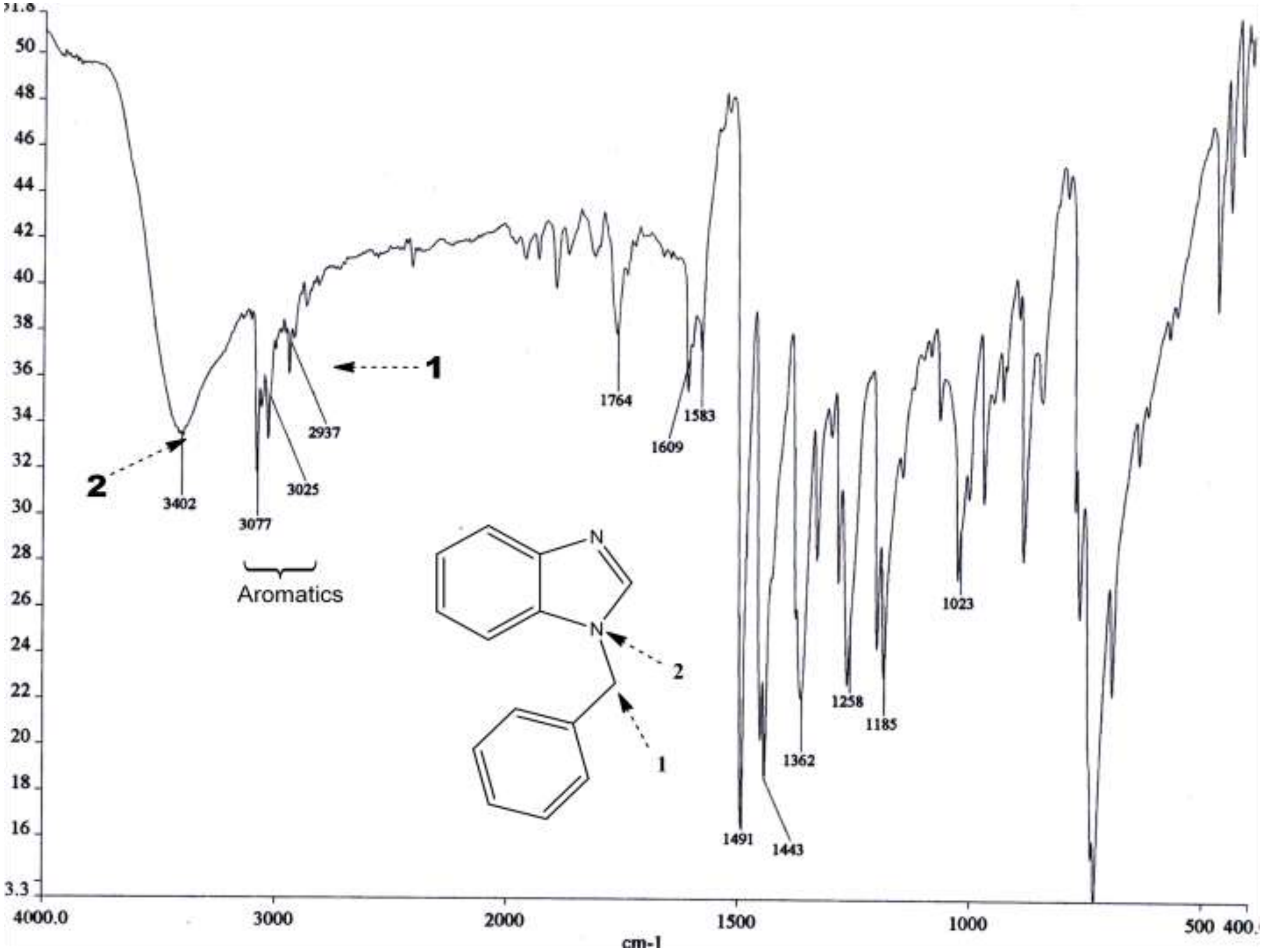

50
48
46
44
42
40
38
36
34
32
30
28
26
24
22
20
18
16
3.3
4000.0
3000
2000
1500
1000
500 400.
cm-1
3402
3077
3025
2937
1764
1609
1583
1491
1443
1362
1258
1185
1023
Aromatics
1
2
N
N

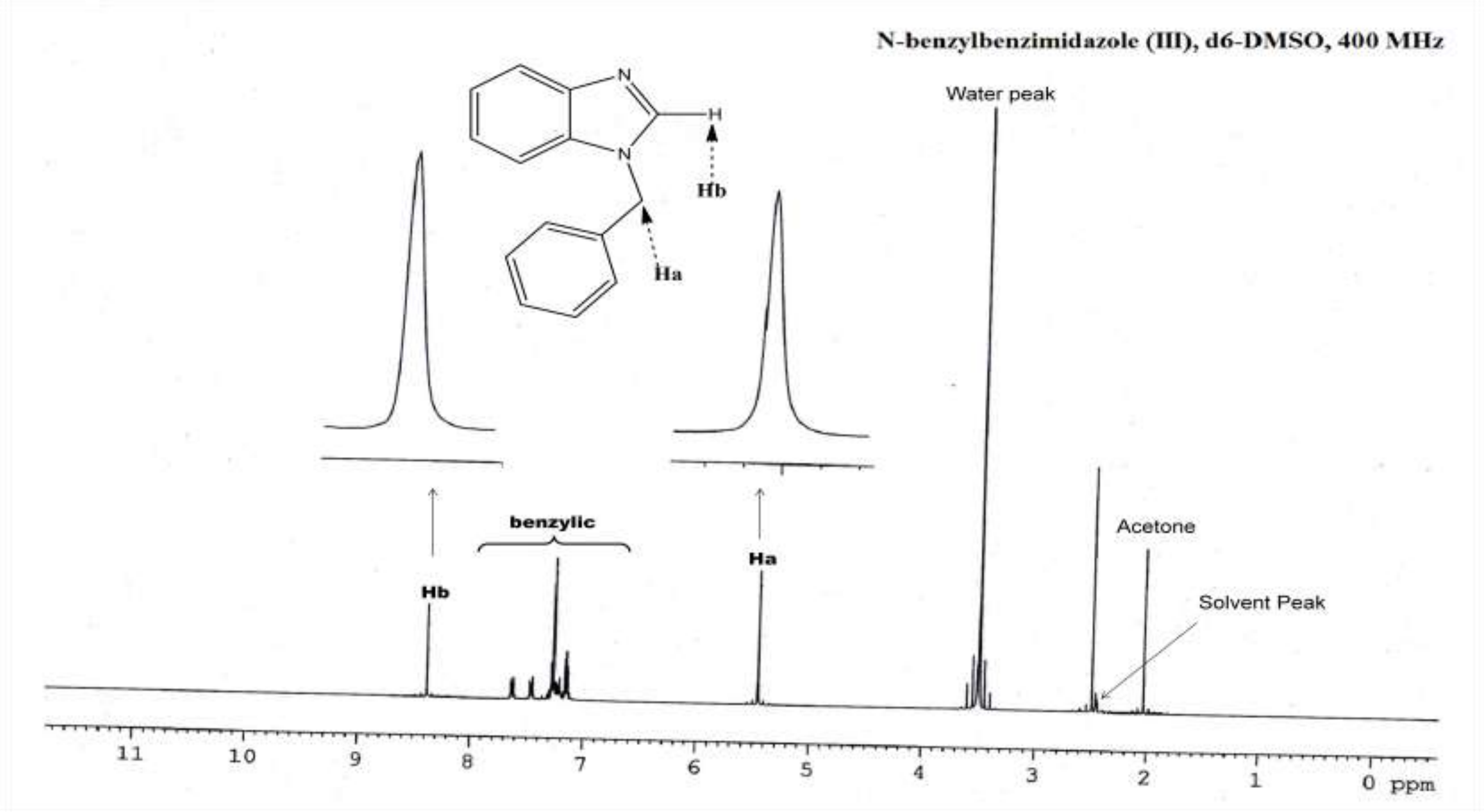
N-benzylbenzimidazole (III), d6-DMSO, 400 MHz
N
N
H
Hb
Ha
Water peak
benzylic
Hb
Ha
Acetone
Solvent Peak
11
10
9
8
7
6
5
4
3
2
1
0 ppm

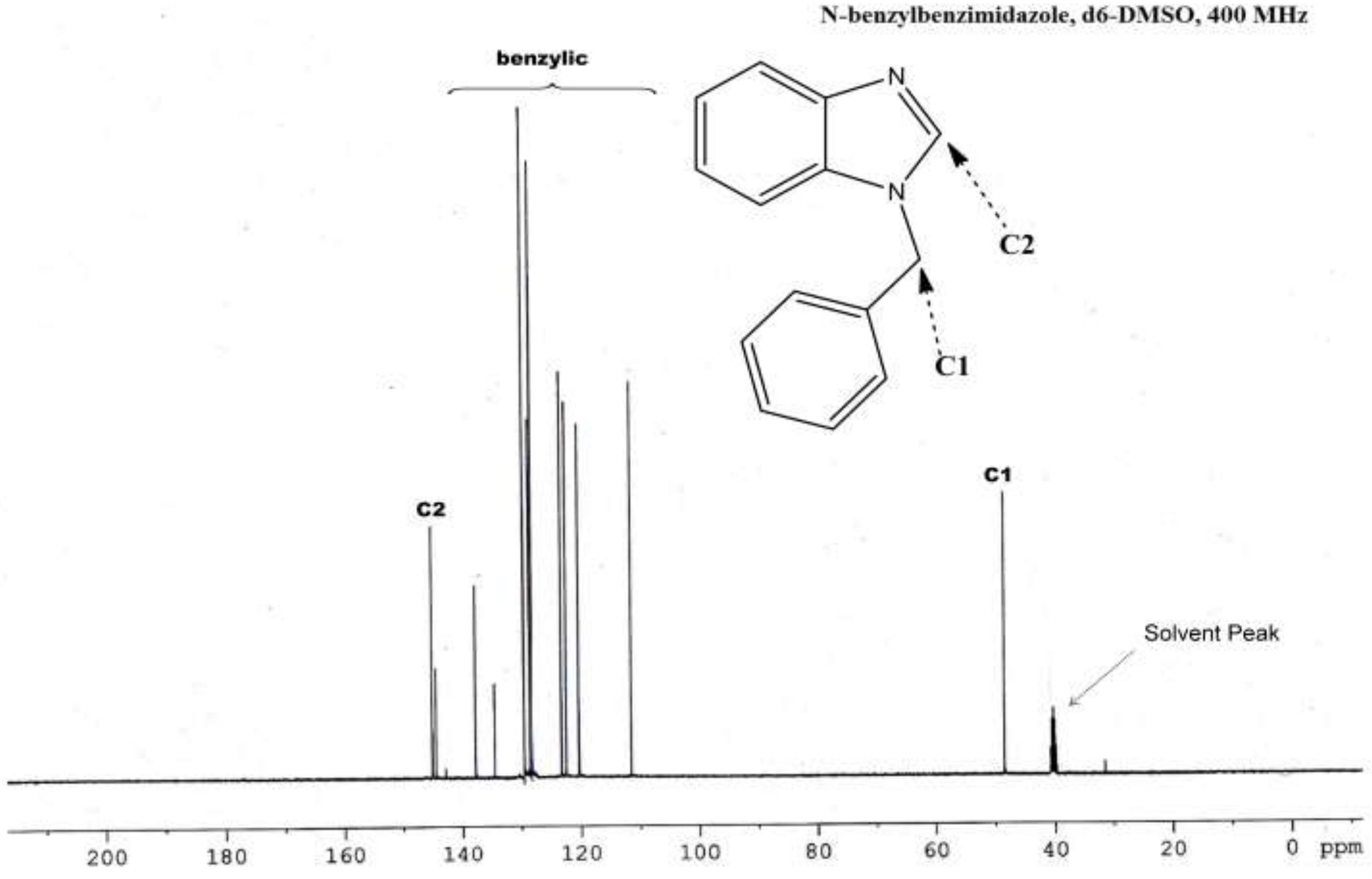
N-benzylbenzimidazole, d6-DMSO, 400 MHz
benzylic
N
N
C2
C1
C2
C1
Solvent Peak
200
180
160
140
120
100
80
60
40
20
0 ppm

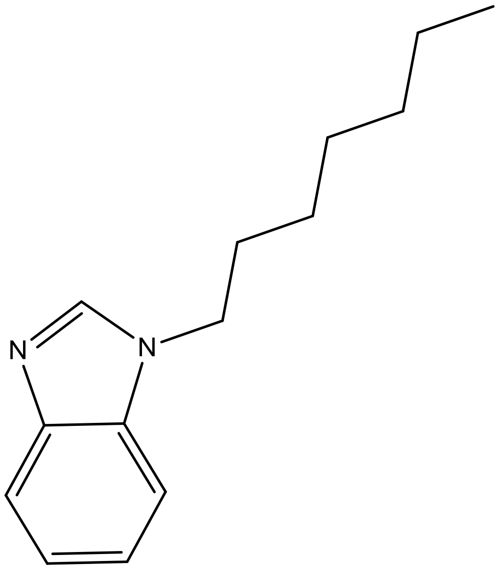

N-heptylbenzimidazole (**V**)

1. FT-IR
2. ^{1}HNMR
3. ^{13}CNMR

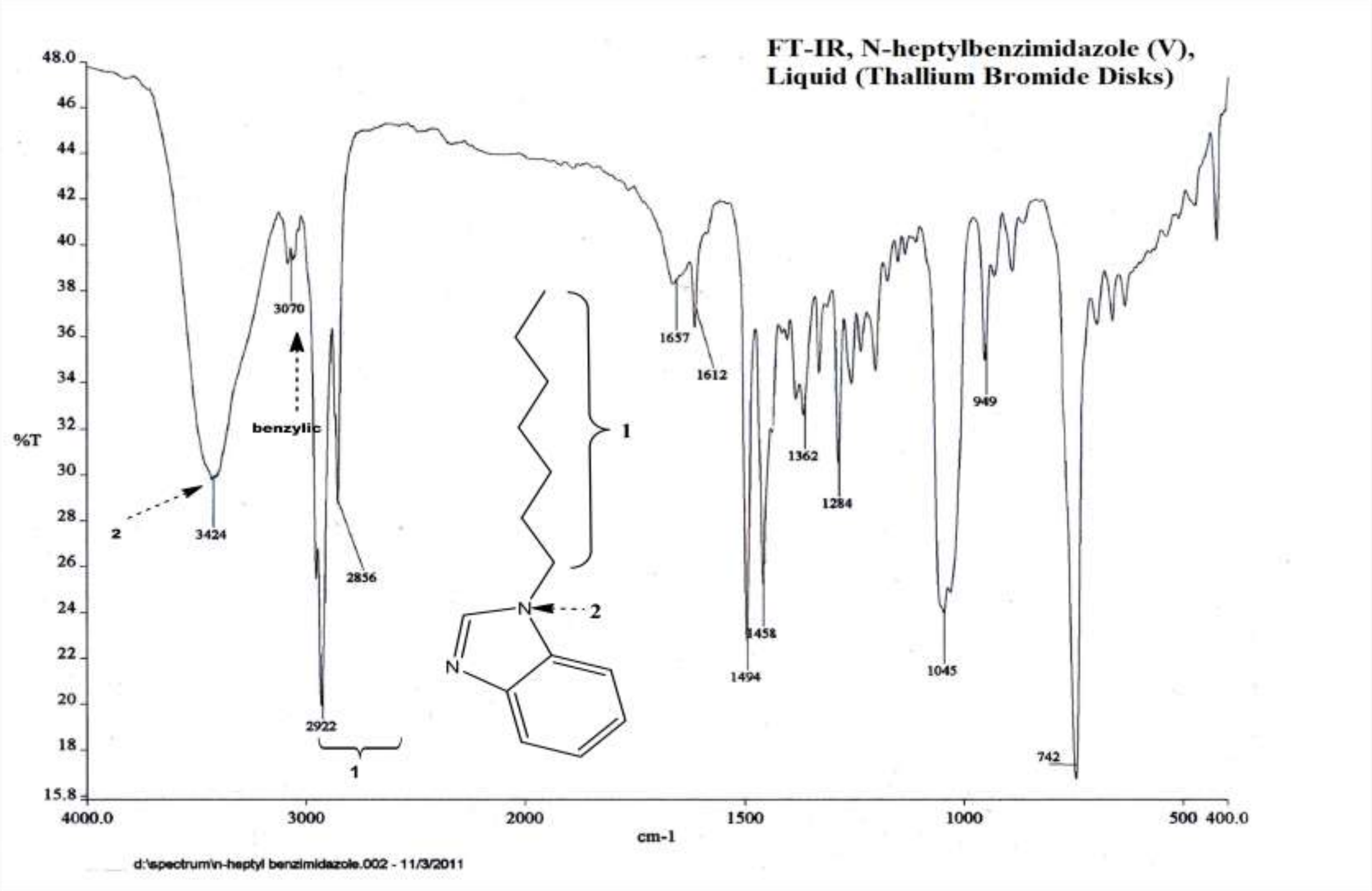

FT-IR, N-heptylbenzimidazole (V),
Liquid (Thallium Bromide Disks)
%T
cm-1
3424
3070
benzylic
2922
2856
1657
1612
1494
1458
1362
1284
1045
949
742
1
2
N
d:\spectrum\n-heptyl benzimidazole.002 - 11/3/2011

N-heptylbenzimidazole, d3-Acetonitril, 400 MHz

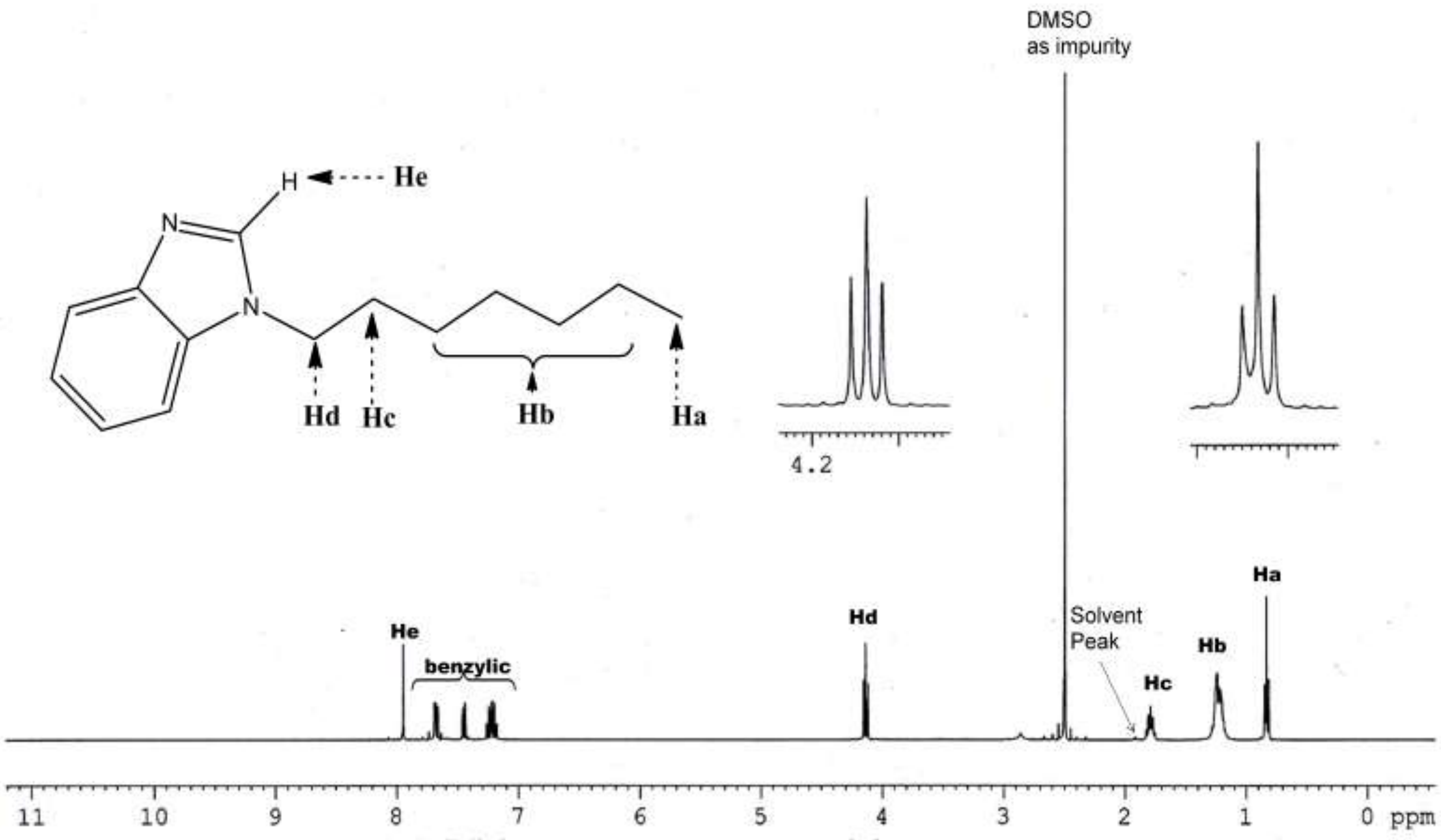

N-heptylbenzimidazole (V), d3-Acetonitril, 400 MHz

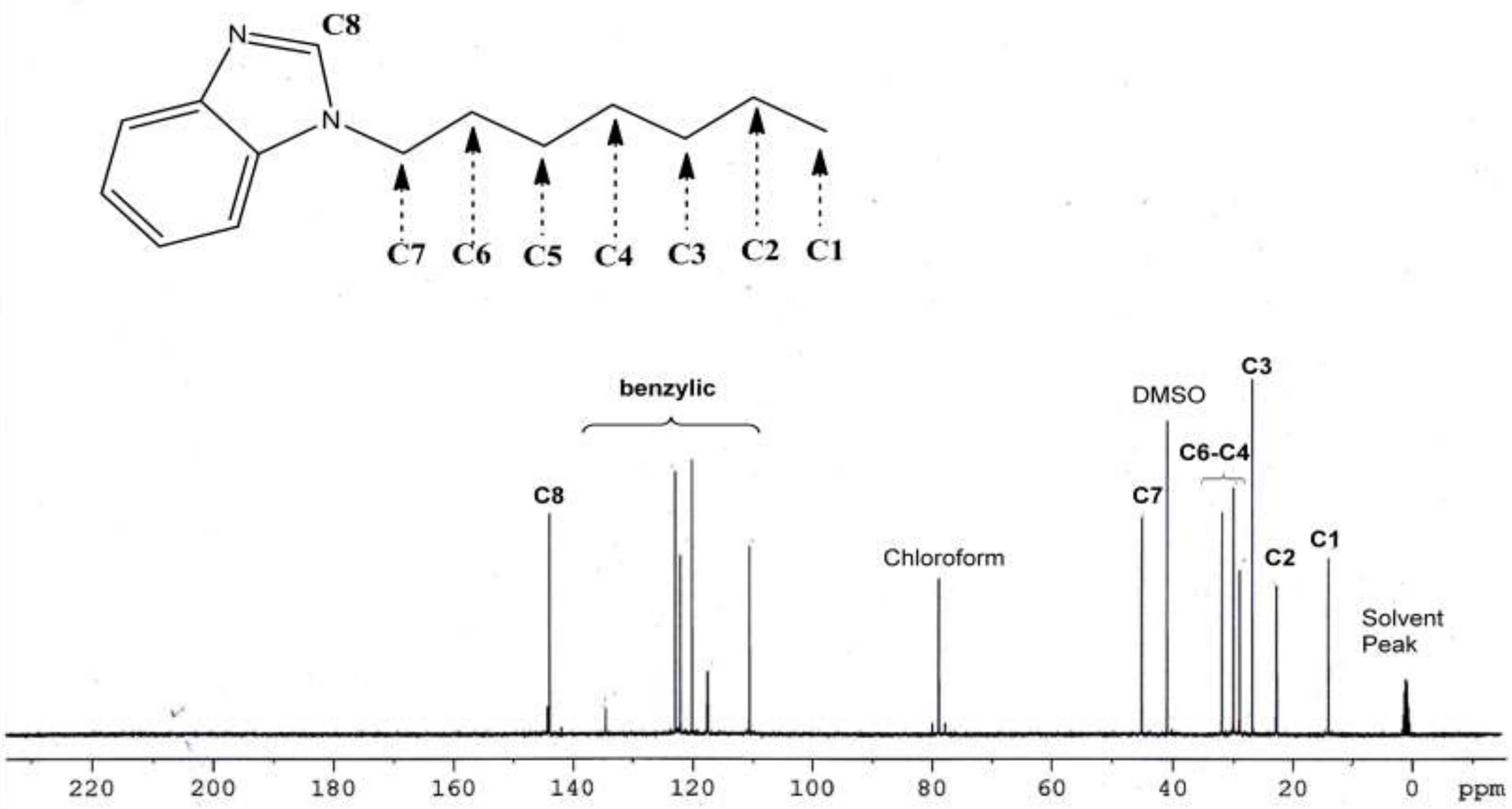

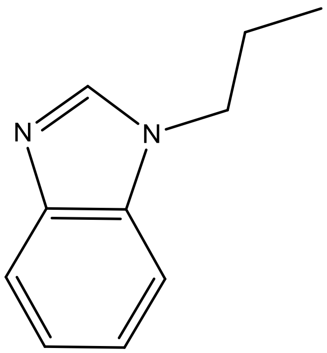

N-propylbenzimidazole (**VI**)

1. FT-IR benzimidazole
2. FT-IR
3. ^{1}HNMR of propyl bromide
4. ^{1}HNMR
5. ^{13}CNMR

FT-IR, Benzimidazole
Solid (KBr)

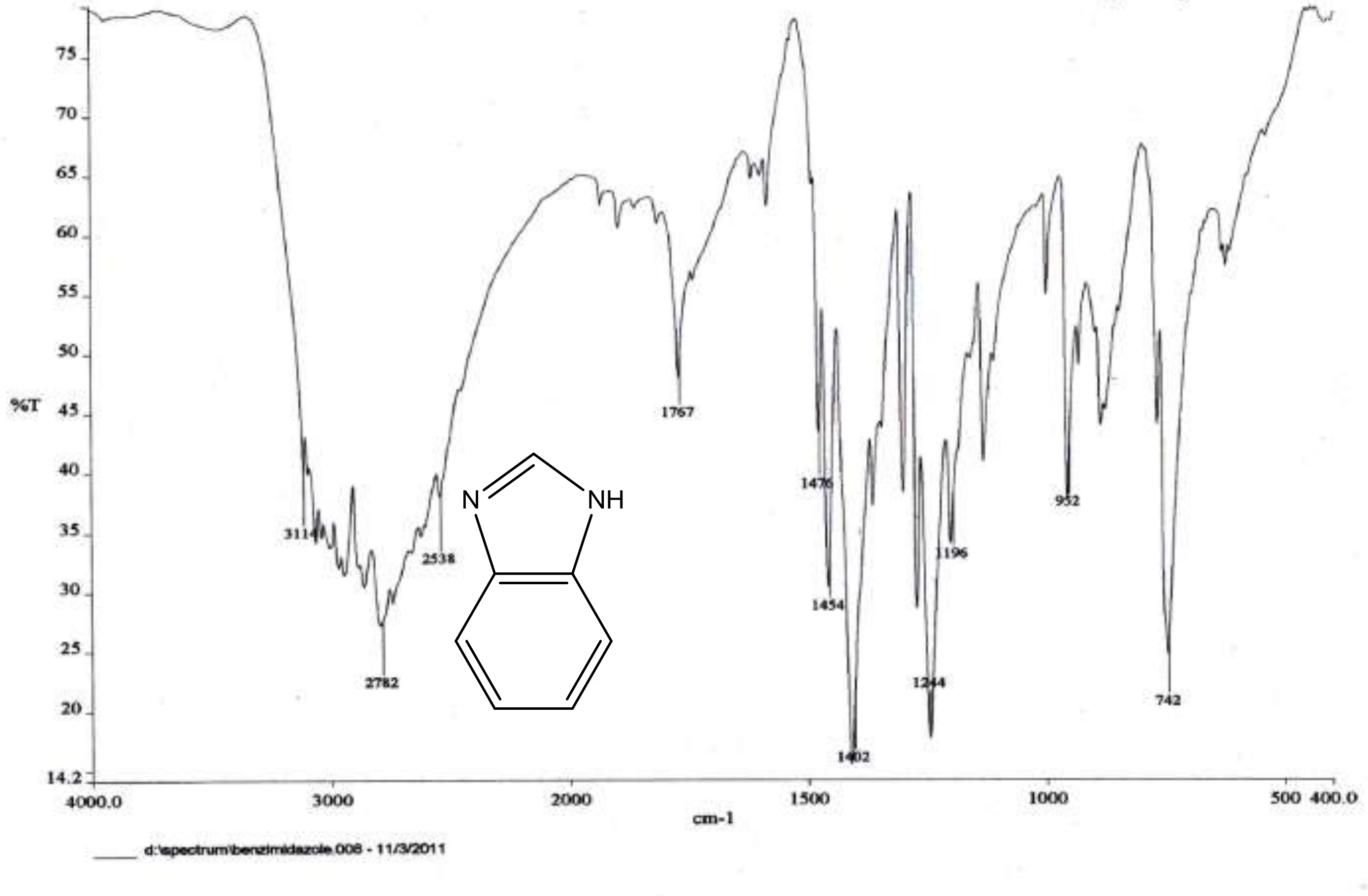

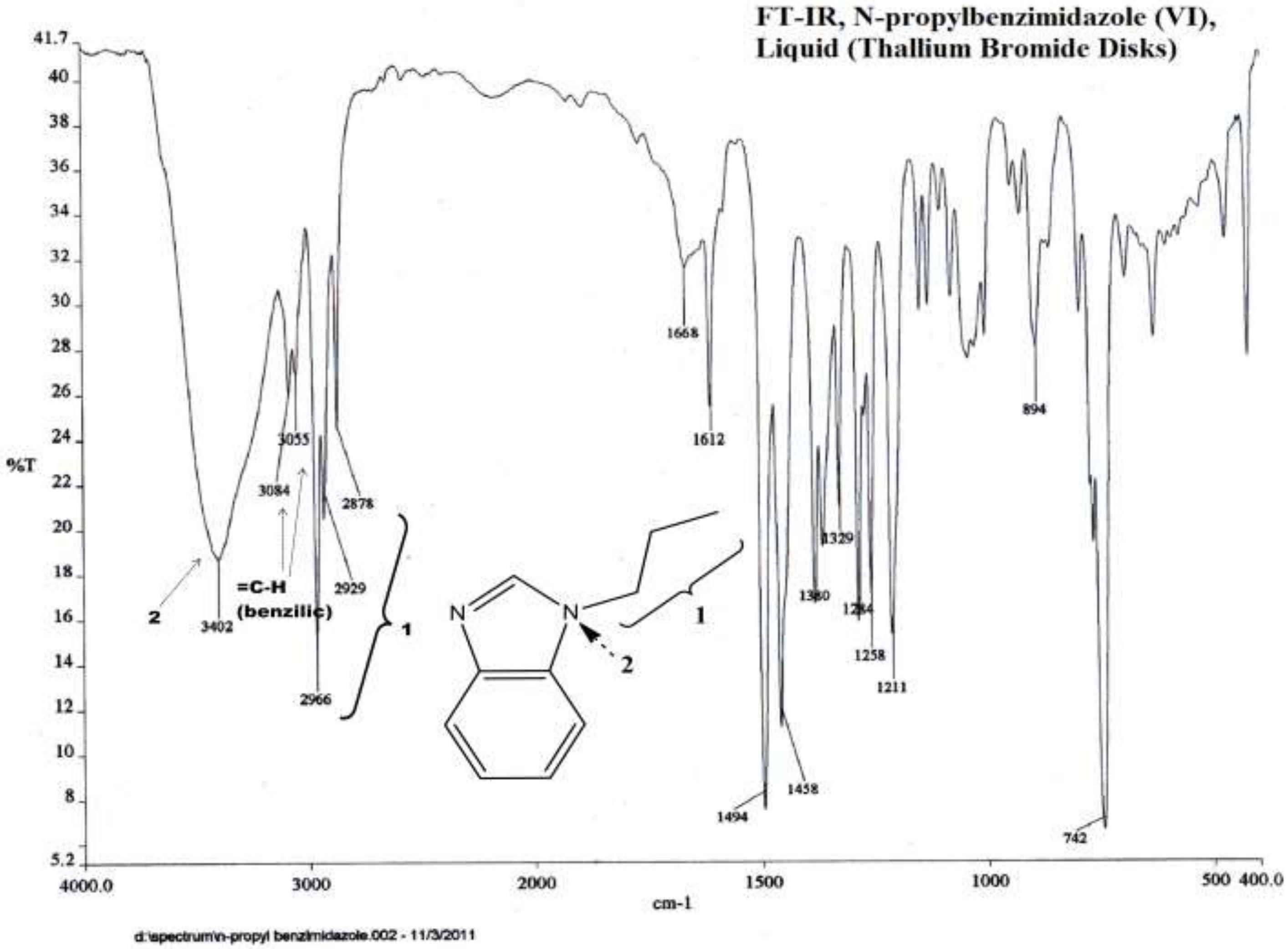

FT-IR, N-propylbenzimidazole (VI),
Liquid (Thallium Bromide Disks)
%T
cm-1
41.7
40
38
36
34
32
30
28
26
24
22
20
18
16
14
12
10
8
5.2
4000.0
3000
2000
1500
1000
500
400.0
3402
3084
3055
2966
2929
2878
=C-H
(benzilic)
2
1
N
N
1668
1612
1494
1458
1380
1329
1284
1258
1211
894
742
d:\spectrum\n-propyl benzimidazole.002 - 11/3/2011

Propyl Bromide, d3-Acetonitrile, 300 MHz

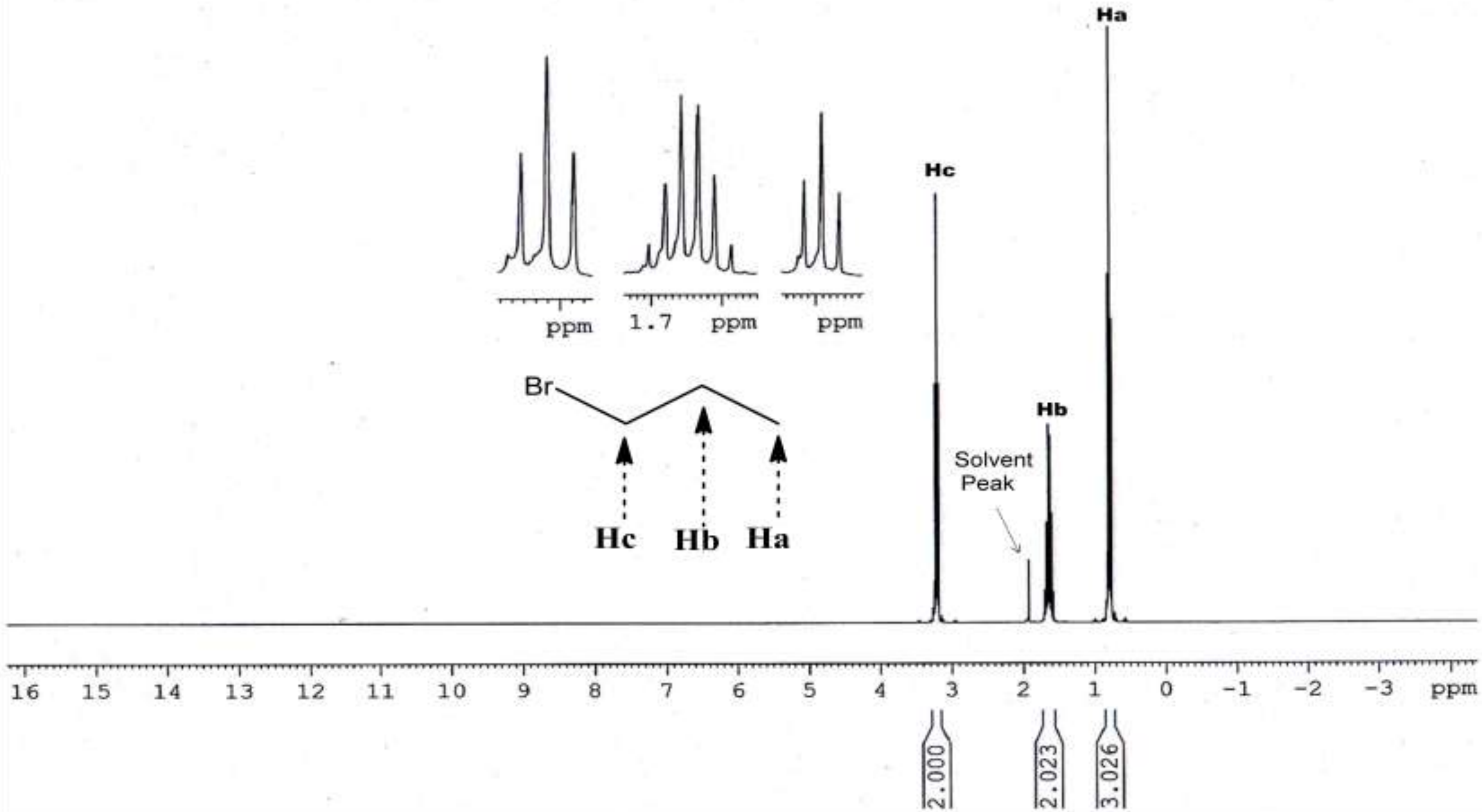

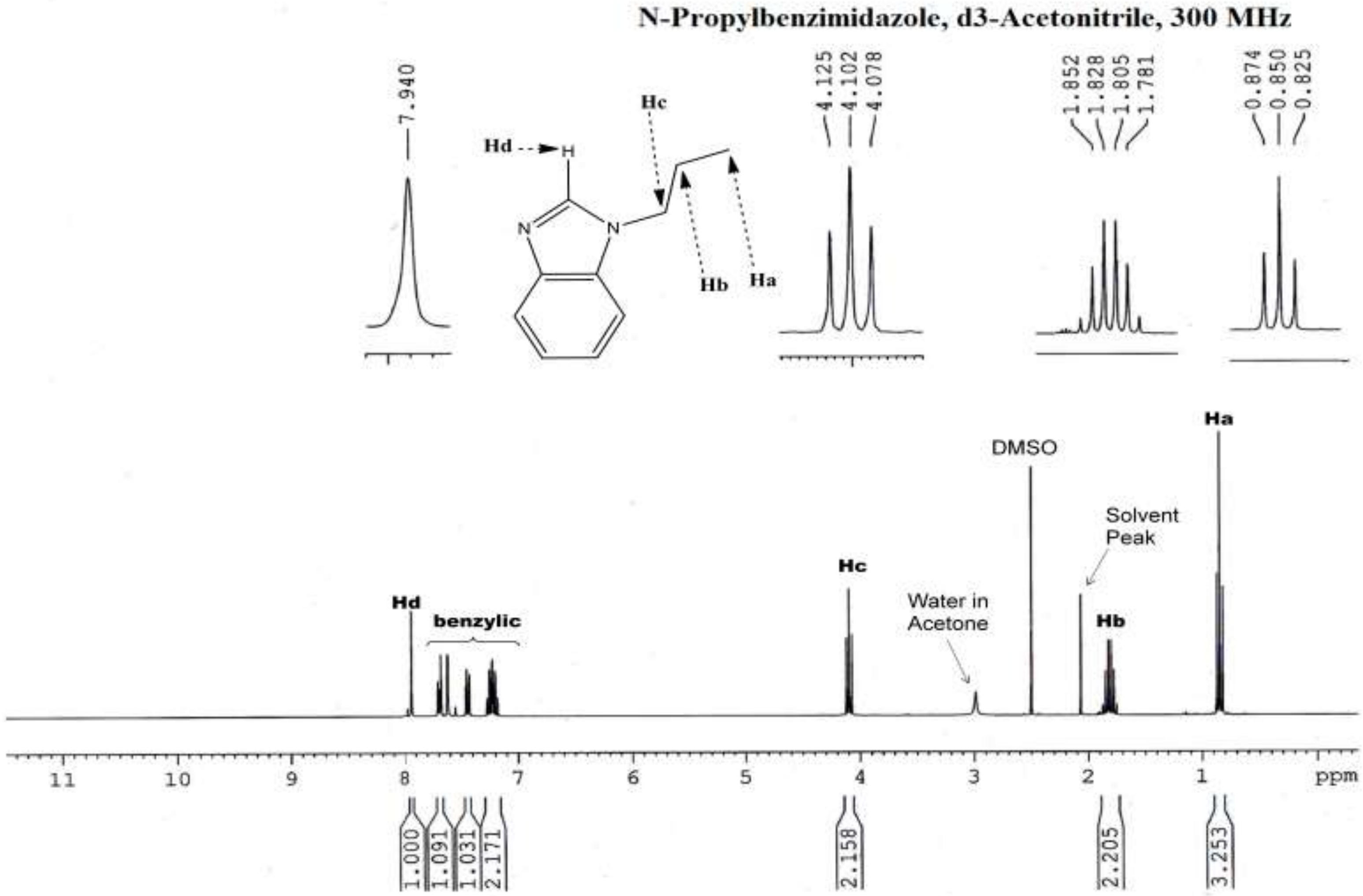

N-Propylbenzimidazole, d3-Acetonitrile, 300 MHz
7.940
Hd
Hc
Hb
Ha
4.125
4.102
4.078
1.852
1.828
1.805
1.781
0.874
0.850
0.825
DMSO
Solvent Peak
Water in Acetone
benzylic
11
10
9
8
7
6
5
4
3
2
1
ppm
1.000
1.091
1.031
2.171
2.158
2.205
3.253

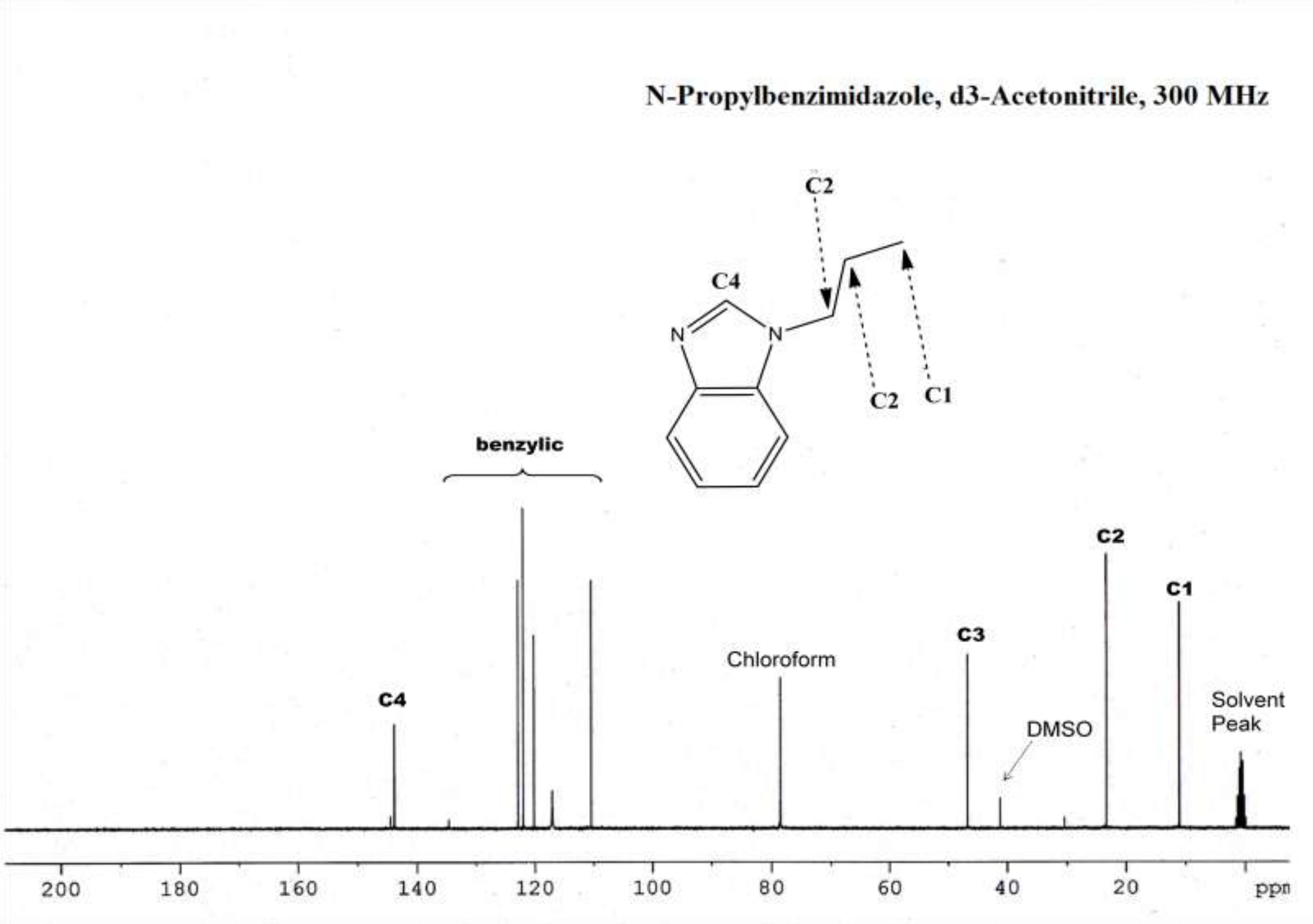
N-Propylbenzimidazole, d3-Acetonitrile, 300 MHz
C2
C4
N
N
C2
C1
benzylic
C2
C1
C3
Chloroform
C4
DMSO
Solvent
Peak
200
180
160
140
120
100
80
60
40
20
ppm

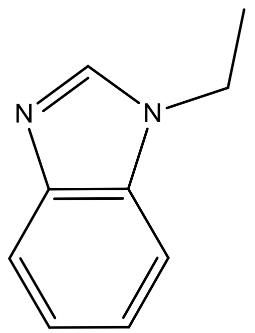

N-ethylbenzimidazole (**VII**)

1. FT-IR
2. ^{1}HNMR
3. ^{13}CNMR

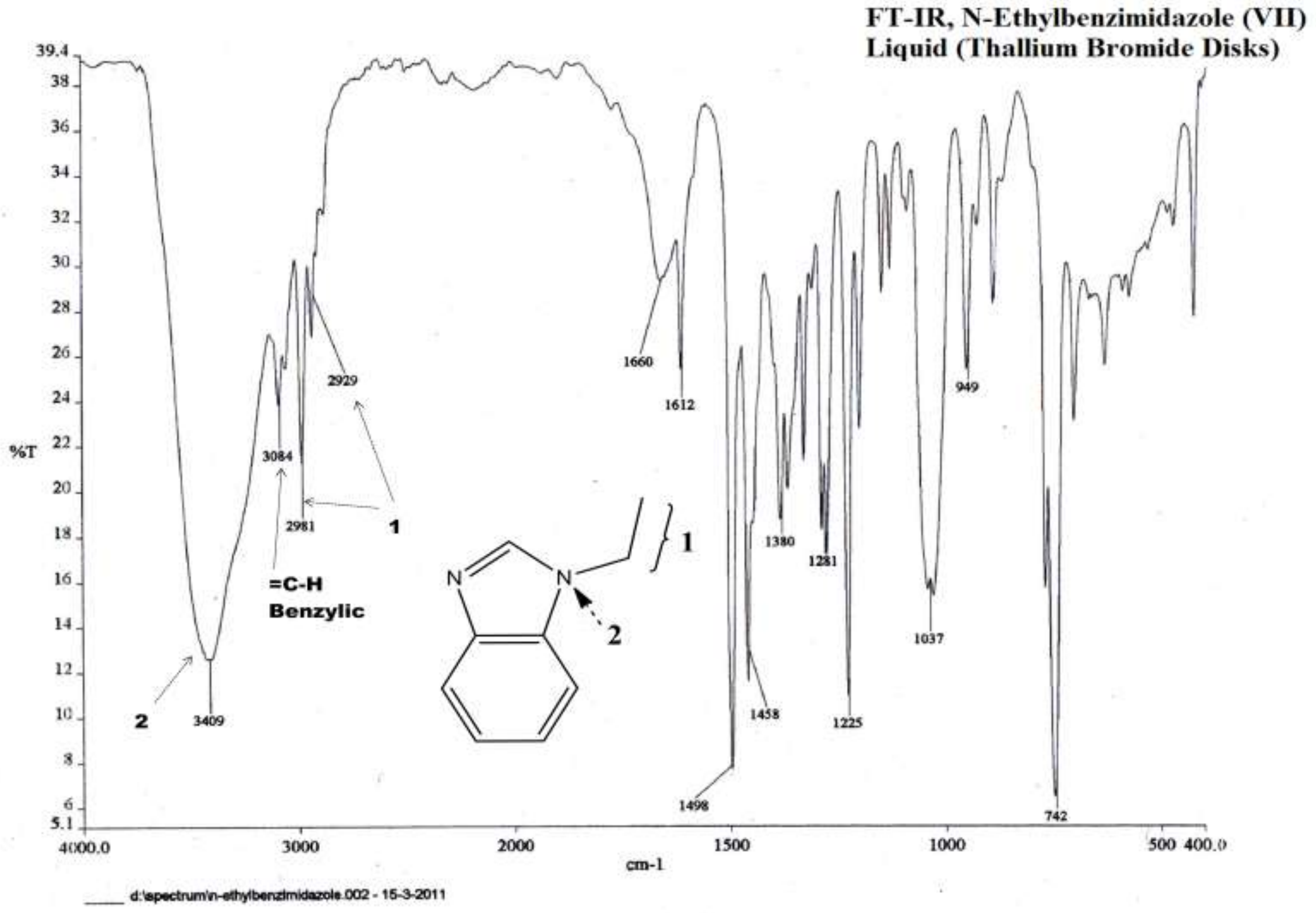
FT-IR, N-Ethylbenzimidazole (VII)
Liquid (Thallium Bromide Disks)
%T
cm-1
3409
3084
2981
2929
1660
1612
1498
1458
1380
1281
1225
1037
949
742
=C-H
Benzylic
1
2
N
N
d:\spectrum\n-ethylbenzimidazole.002 - 15-3-2011

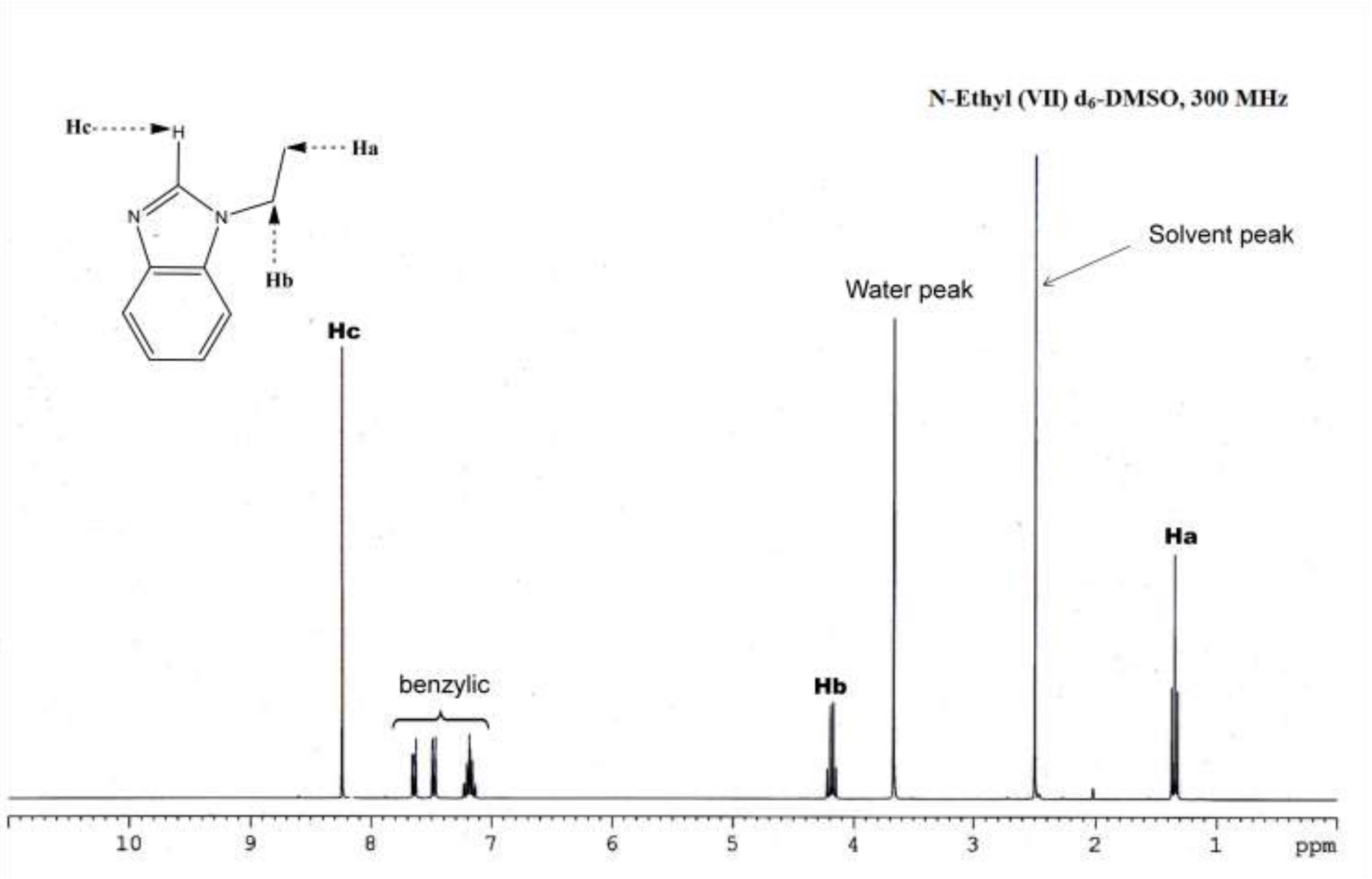
N-Ethyl (VII) d₆-DMSO, 300 MHz
Hc
H
Ha
N
N
Hb
Hc
Solvent peak
Water peak
Ha
benzylic
Hb
10
9
8
7
6
5
4
3
2
1
ppm

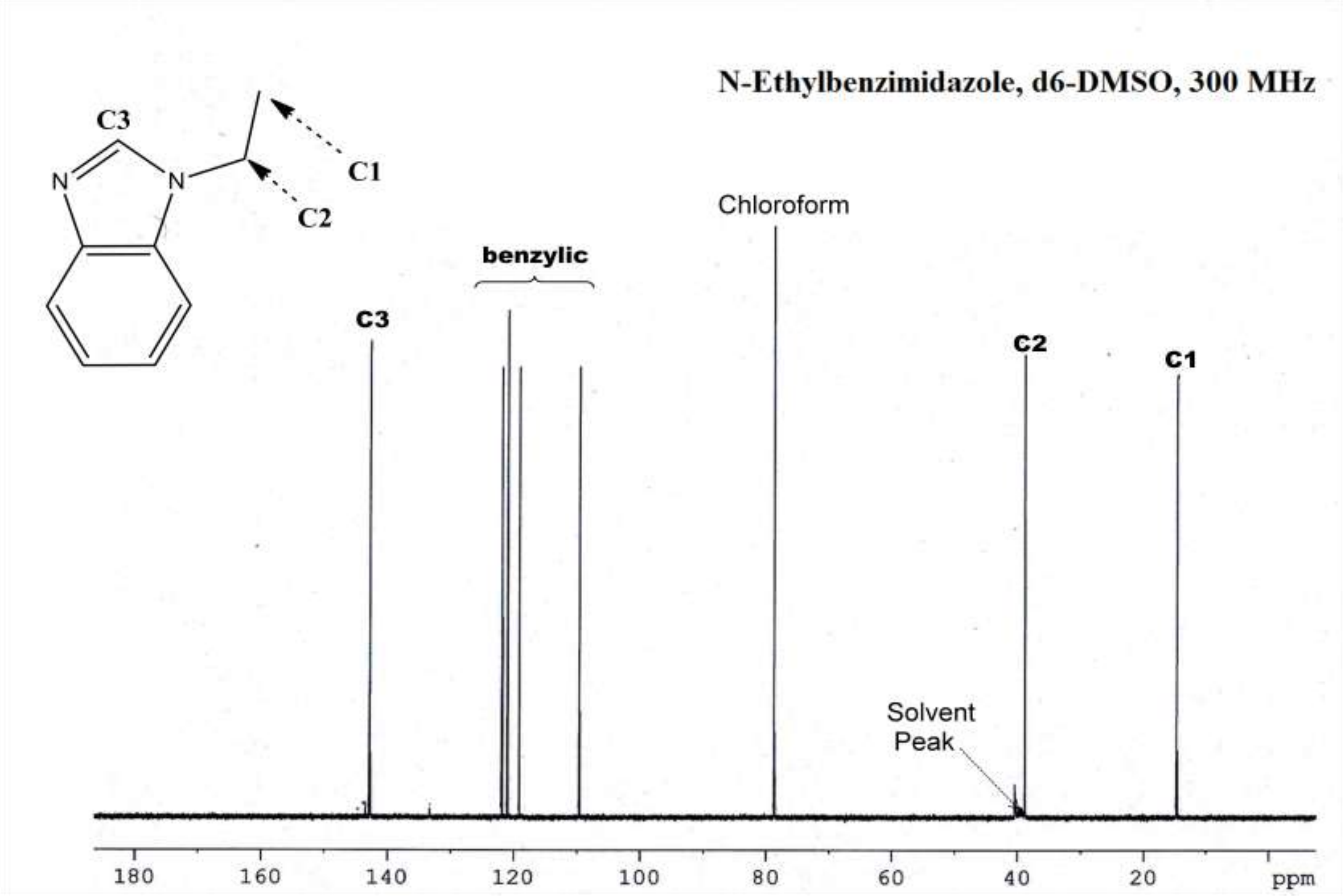

N-Ethylbenzimidazole, d6-DMSO, 300 MHz
C3
N
N
C1
C2
Chloroform
benzylic
C3
C2
C1
Solvent
Peak
180
160
140
120
100
80
60
40
20
ppm

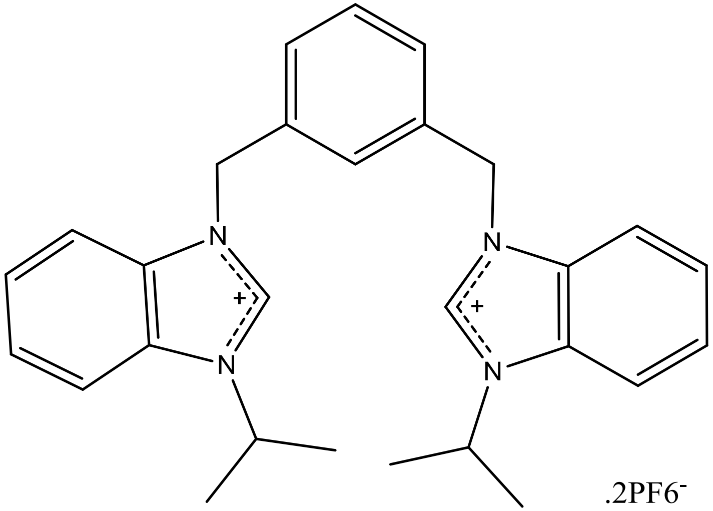

3,3'-(1,3-phenylenebis(methylene))bis(1-isopropyl-1H-benzimidazolium) salt (**VIII.2PF$_6$**)

1. FT-IR
2. ^{1}HNMR
3. ^{13}CNMR

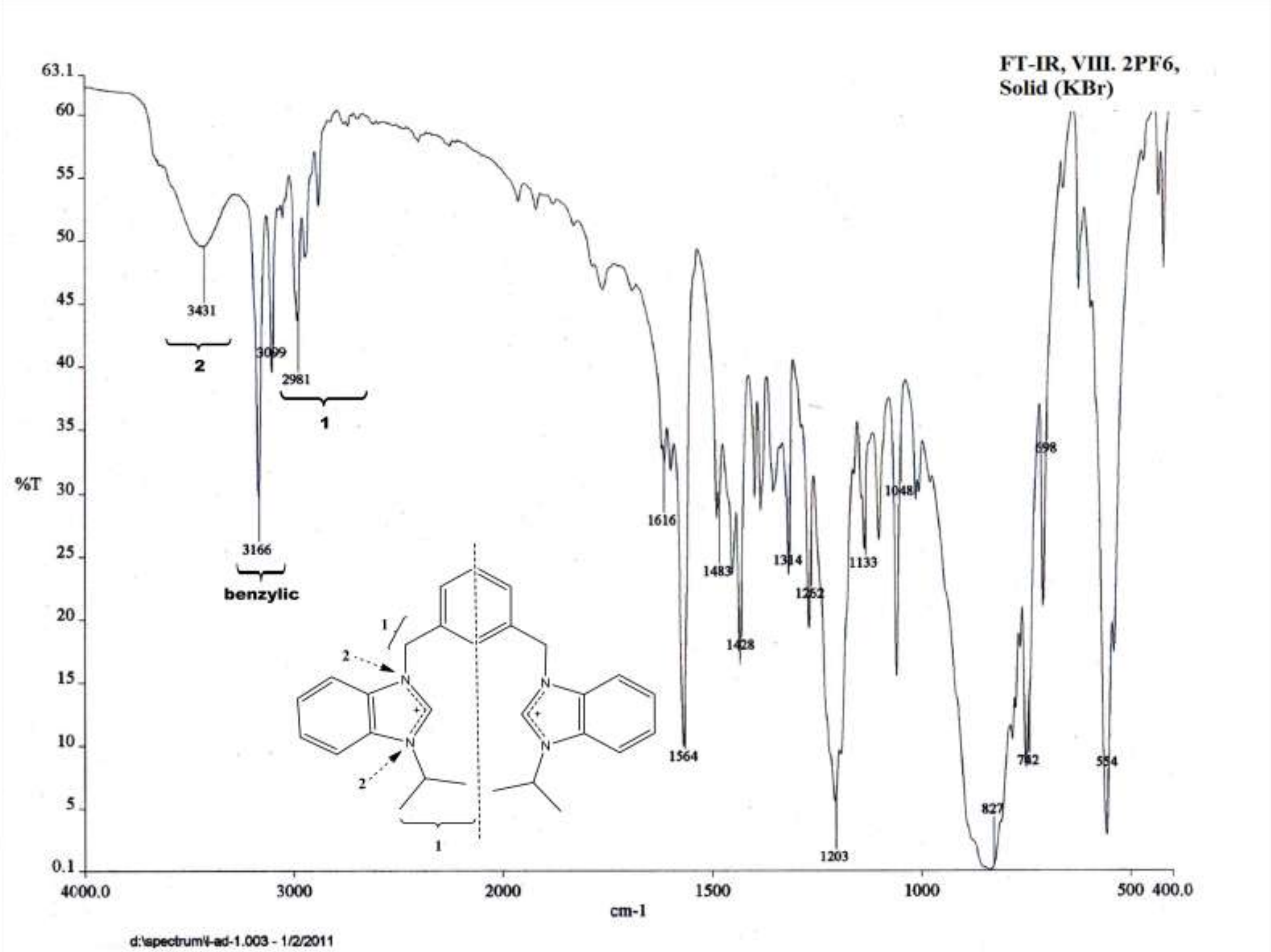
FT-IR, VIII. 2PF6,
Solid (KBr)
%T
cm-1
3431
2
3166
benzylic
3099
2981
1
1616
1564
1483
1428
1314
1262
1203
1133
1048
827
782
698
554
d:\spectrum\i-ad-1.003 - 1/2/2011

VIII. 2PF6, d6-DMSO, 400 Mhz

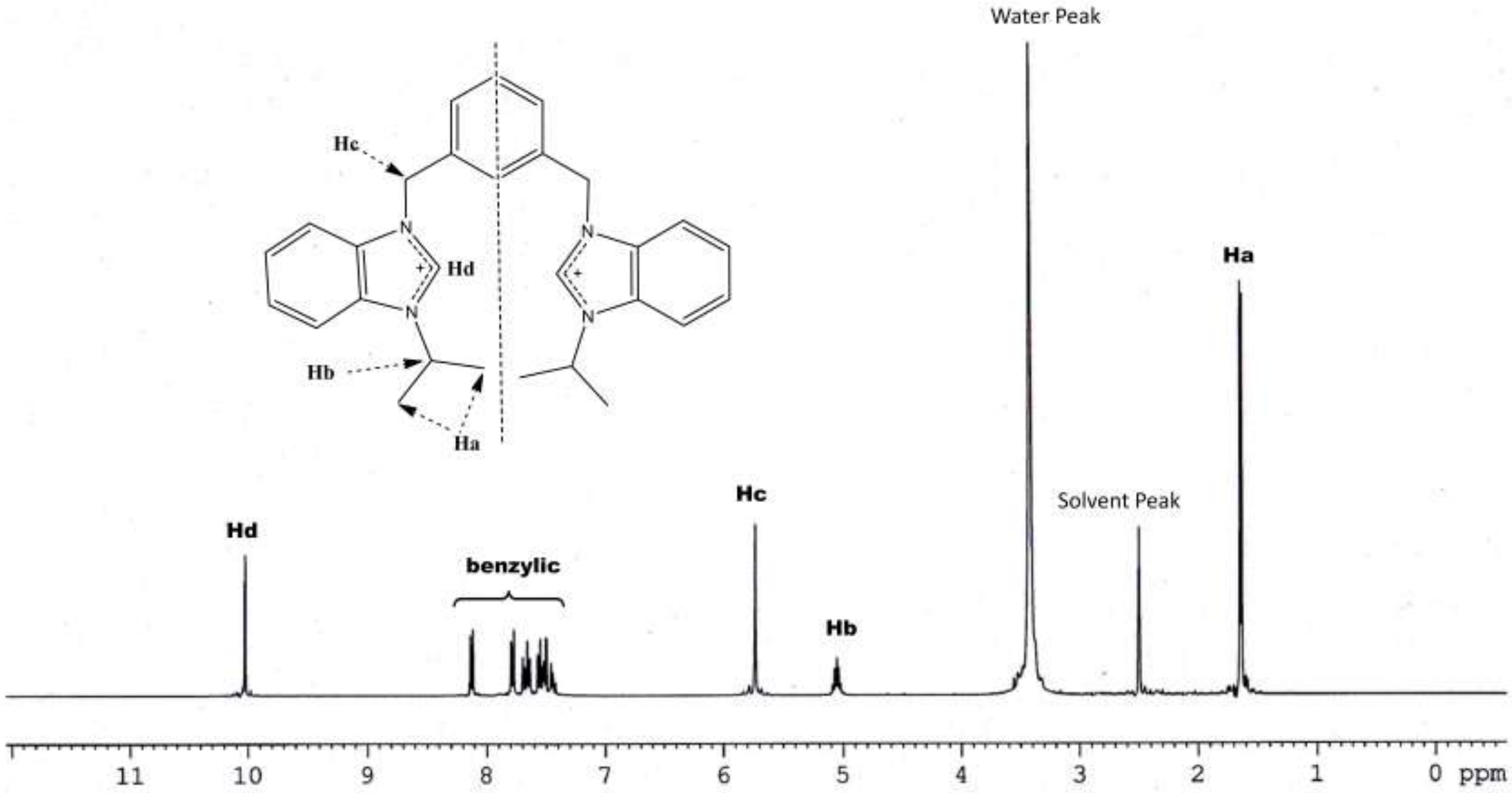

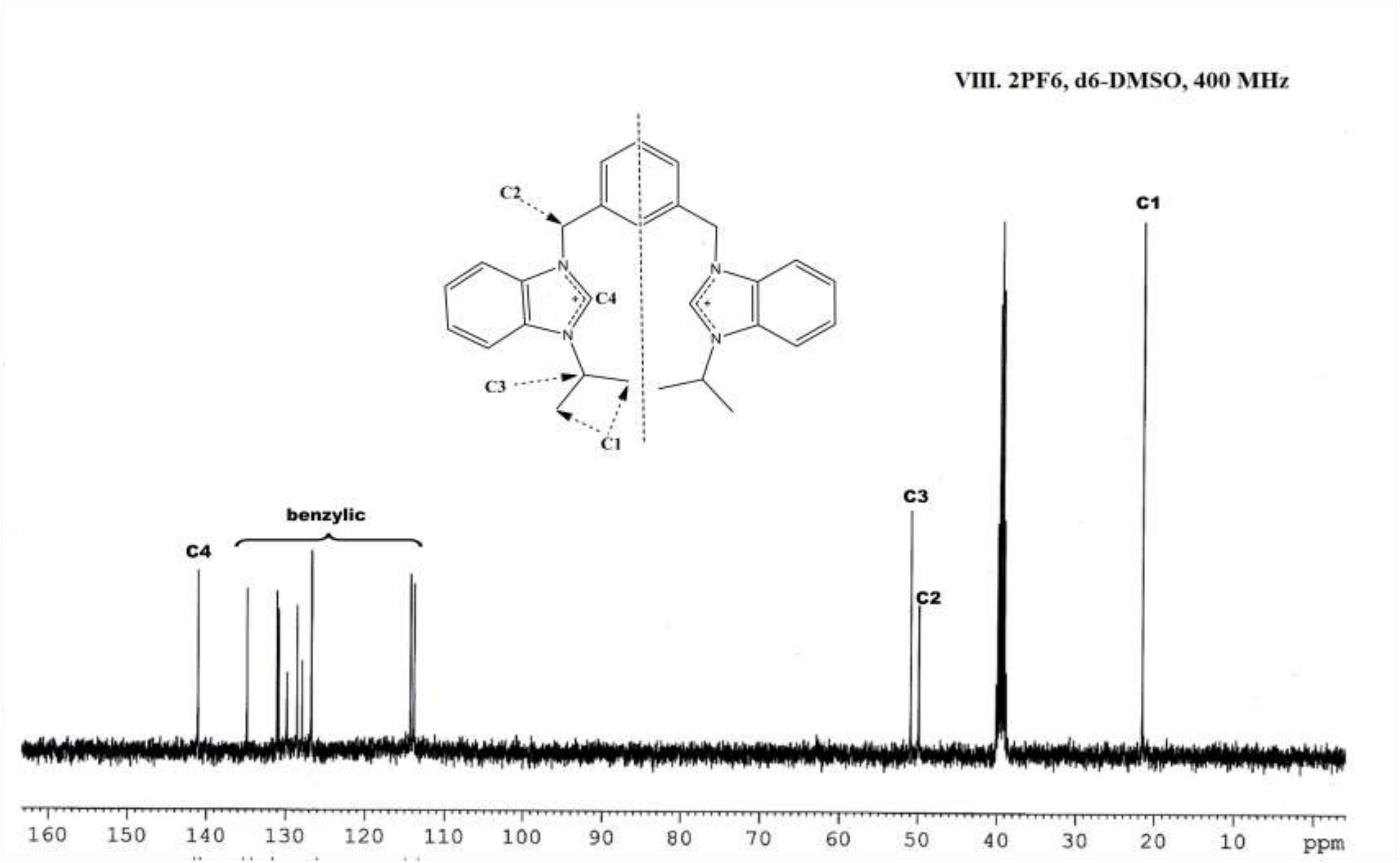
VIII. 2PF6, d6-DMSO, 400 MHz
C2
C4
C3
C1
C1
C3
C2
benzylic
C4
160 150 140 130 120 110 100 90 80 70 60 50 40 30 20 10 ppm

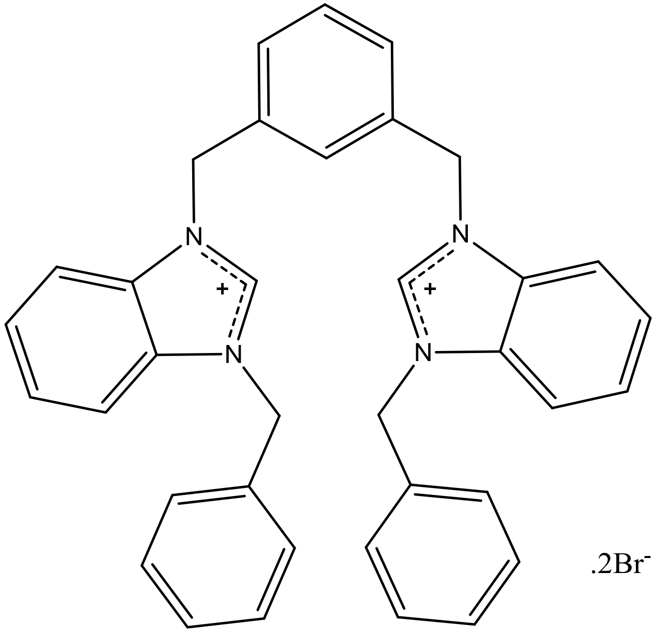

3,3'-(1,3-phenylenebis(methylene))bis(1-benzyl-1H-benzimidazolium) salt (**X.2Br**)

1. FT-IR
2. ^{1}HNMR
3. ^{13}CNMR

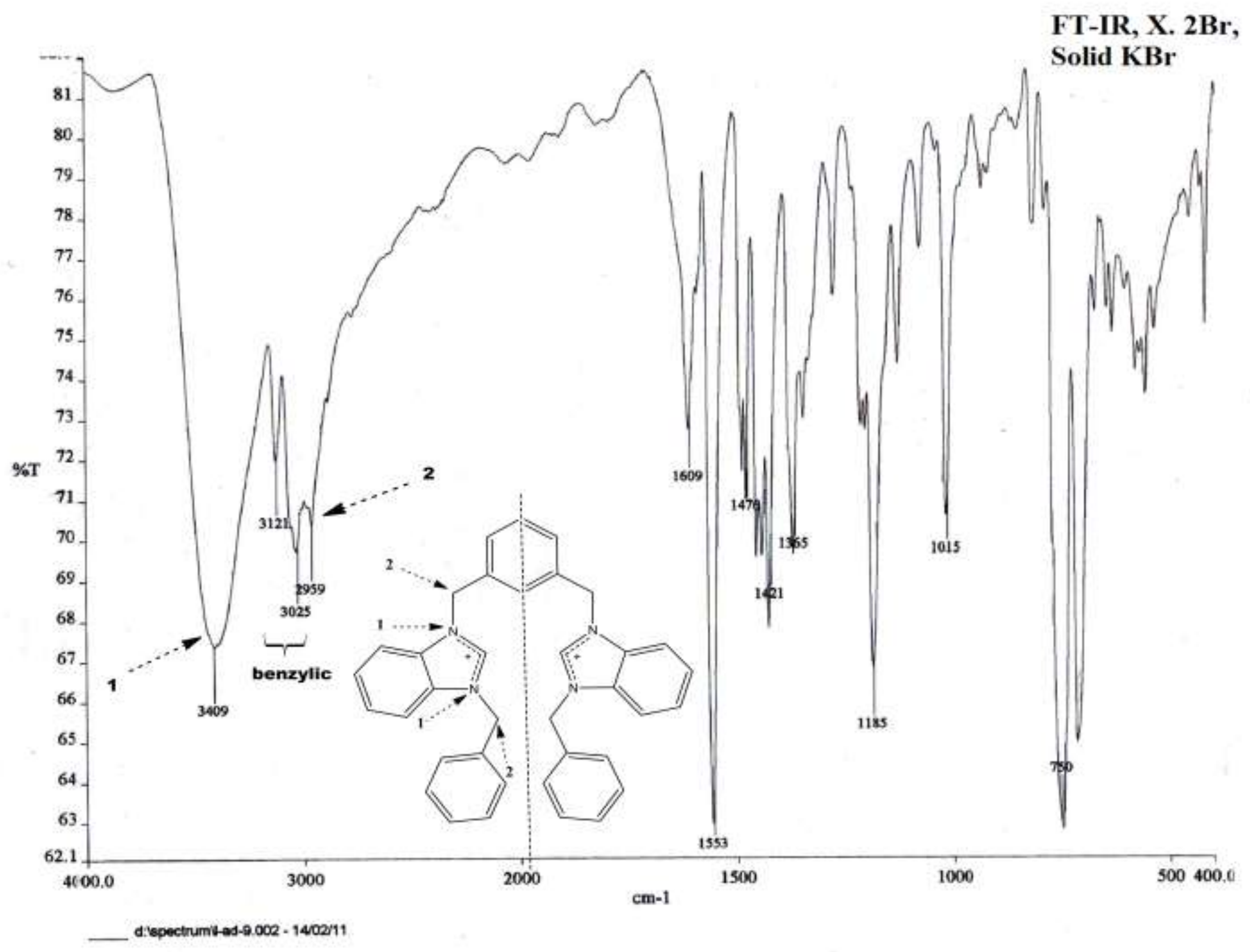

FT-IR, X. 2Br,
Solid KBr
%T
cm-1
3409
3121
3025
2959
benzylic
1609
1476
1365
1421
1553
1185
1015
750
d:\spectrum\l-ad-9.002 - 14/02/11

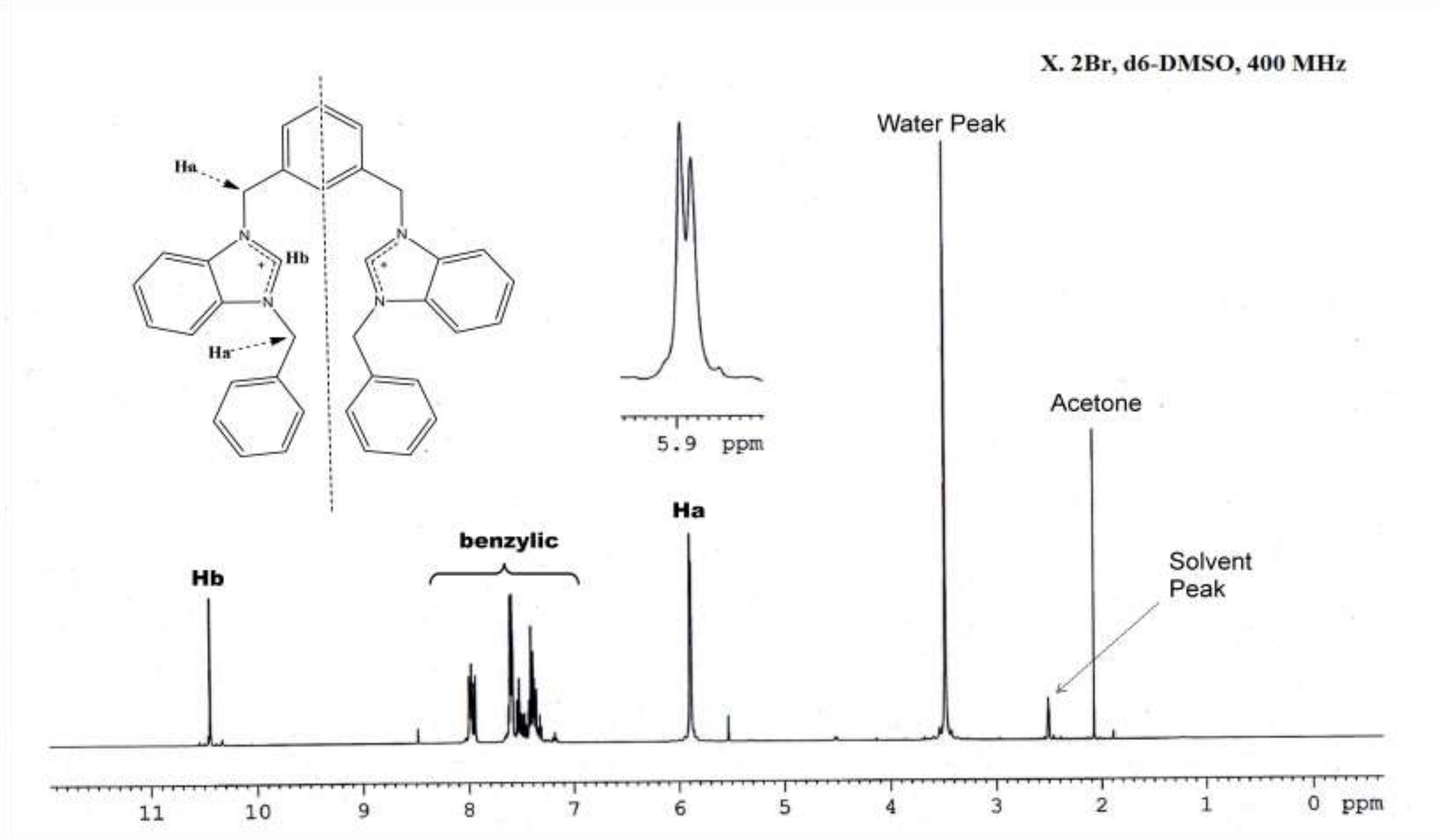
X. 2Br, d6-DMSO, 400 MHz
Ha
Hb
Ha
5.9 ppm
Water Peak
Acetone
Ha
benzylic
Hb
Solvent
Peak
11
10
9
8
7
6
5
4
3
2
1
0 ppm

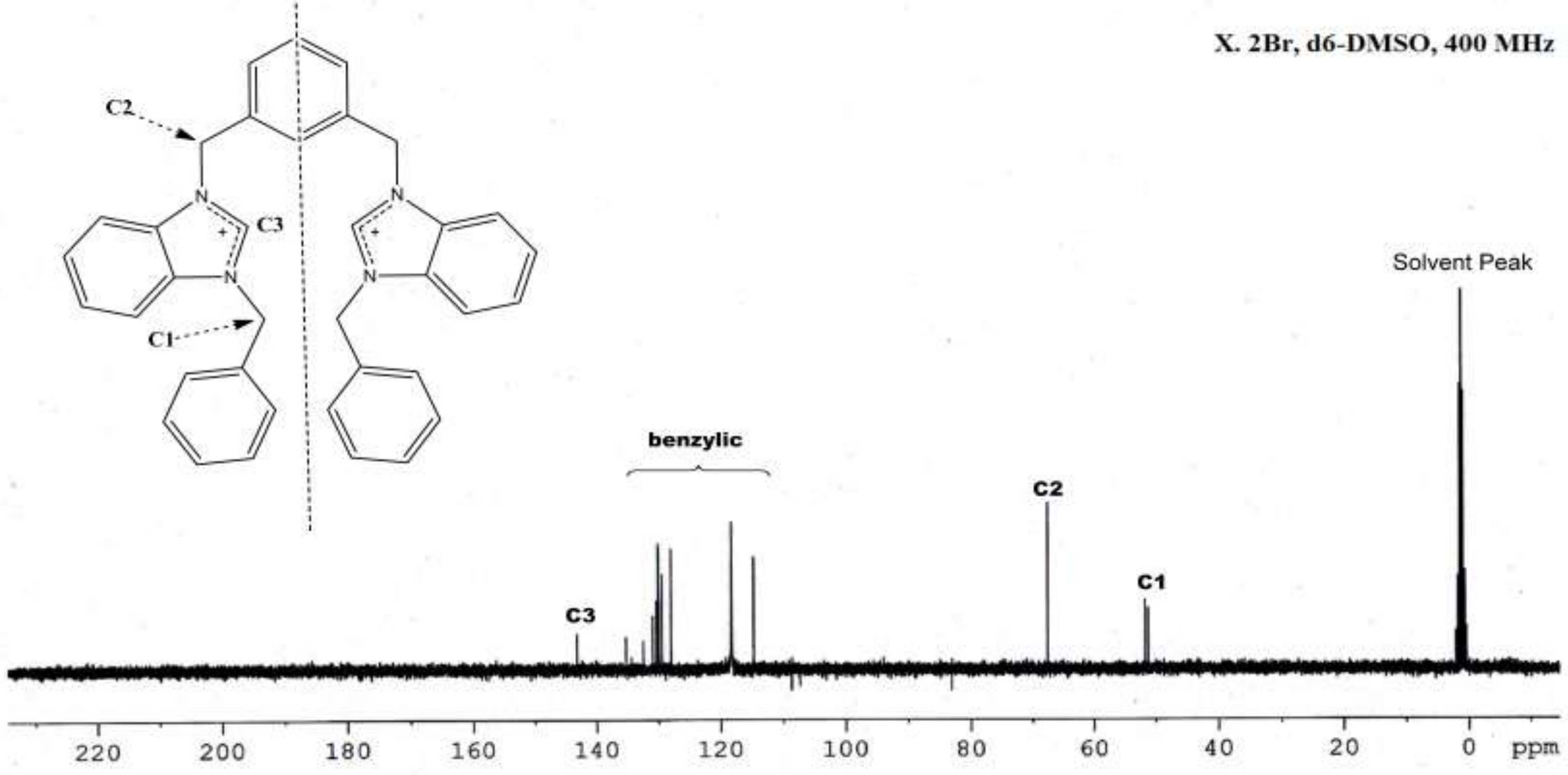

X. 2Br, d6-DMSO, 400 MHz
C2
C3
C1
N
Solvent Peak
benzylic
C2
C1
C3
220
200
180
160
140
120
100
80
60
40
20
0
ppm

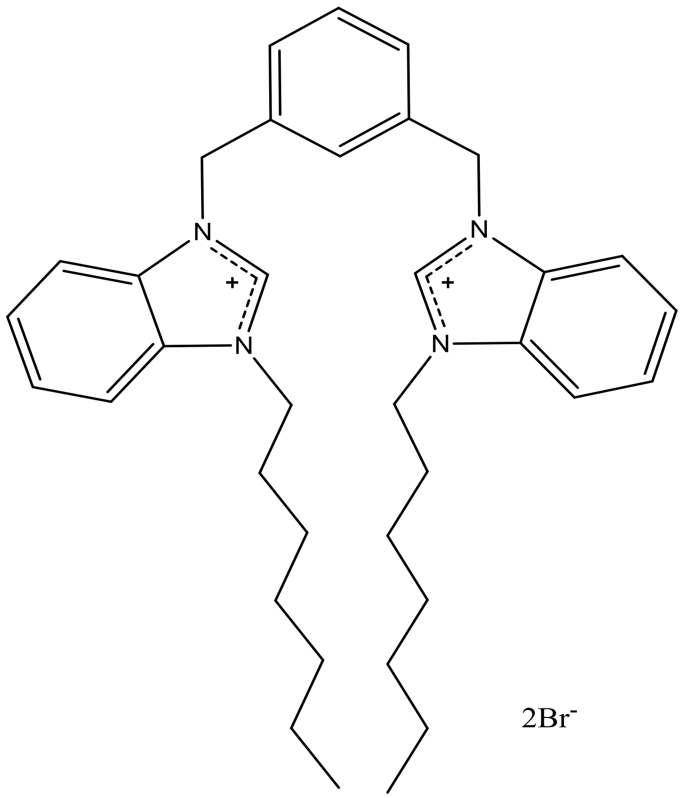

3,3'-(1,3-phenylenebis(methylene))bis(1-heptyl-1H-benzimidazolium) salt (**XII.2Br**)

1. FT-IR
2. ^{1}HNMR
3. ^{13}CNMR

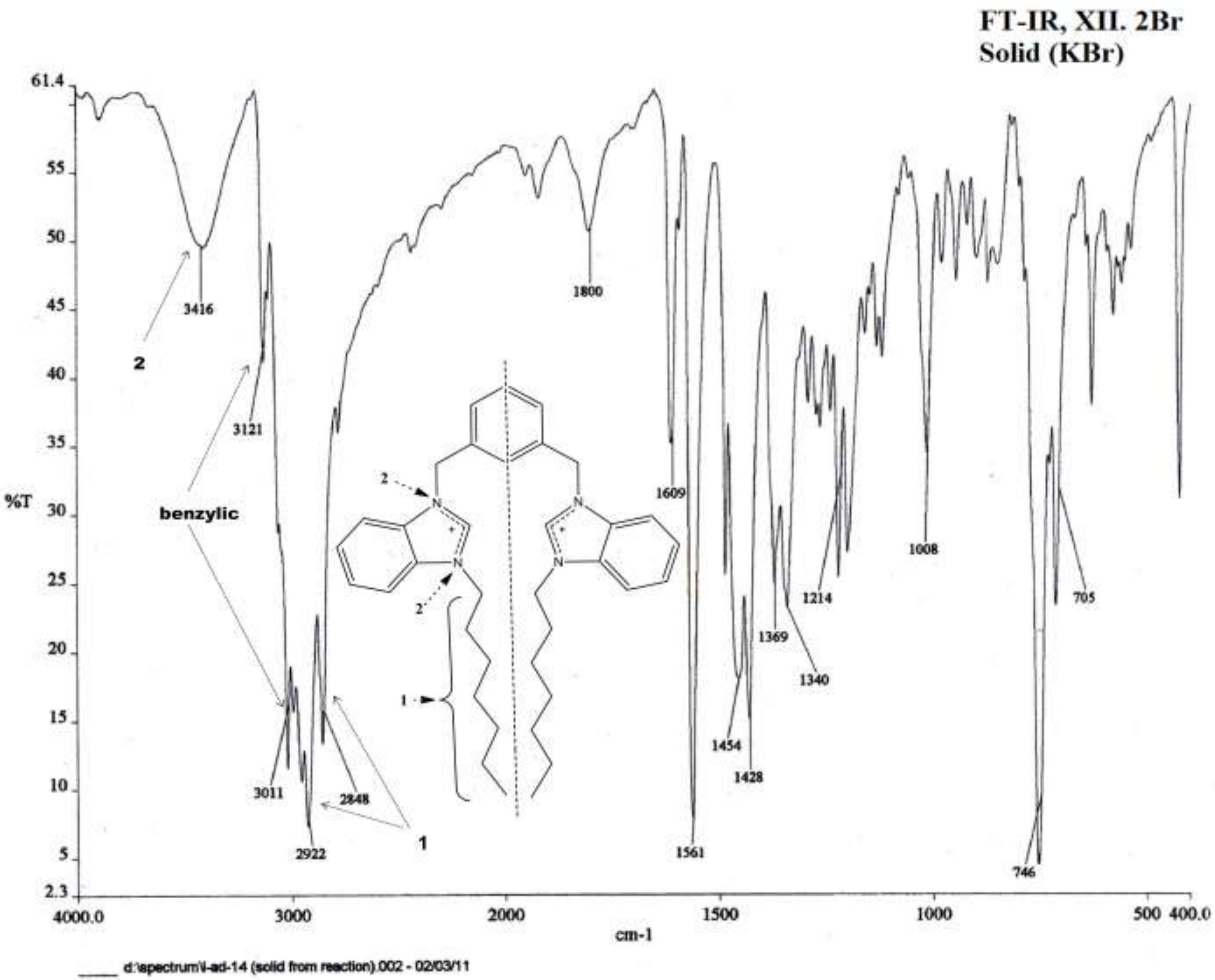
FT-IR, XII. 2Br
Solid (KBr)
61.4
55
50
45
40
35
%T
30
25
20
15
10
5
2.3
4000.0
3000
2000
1500
1000
500
400.0
cm-1
3416
2
3121
benzylic
3011
2922
2848
1
1800
1609
1561
1454
1428
1369
1340
1214
1008
746
705
d:\spectrum\l-ad-14 (solid from reaction).002 - 02/03/11

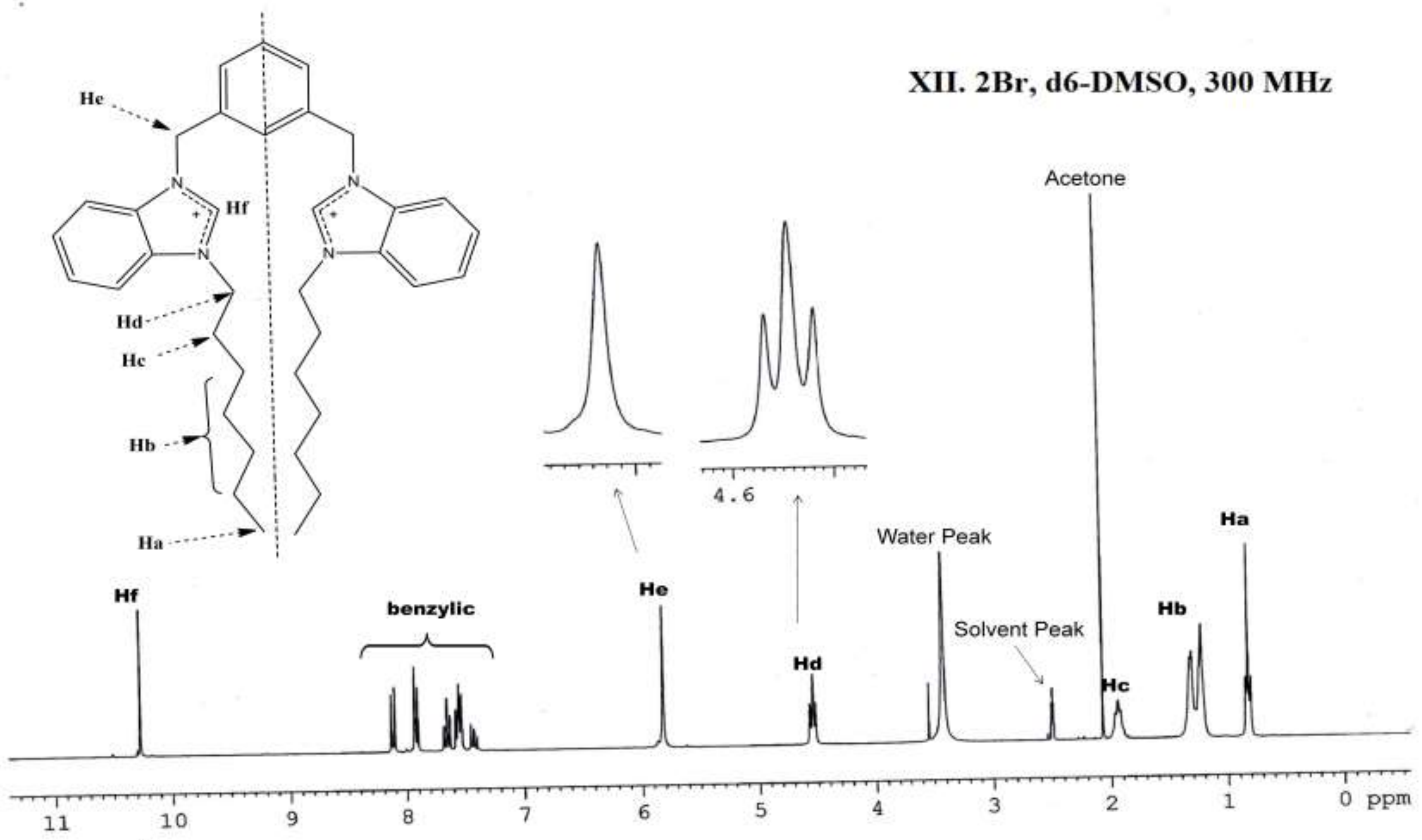
XII. 2Br, d6-DMSO, 300 MHz
He
Hf
Hd
Hc
Hb
Ha
Acetone
Water Peak
Solvent Peak
benzylic
4.6
11
10
9
8
7
6
5
4
3
2
1
0 ppm

XII. 2Br, d6-DMSO, 300 MHz

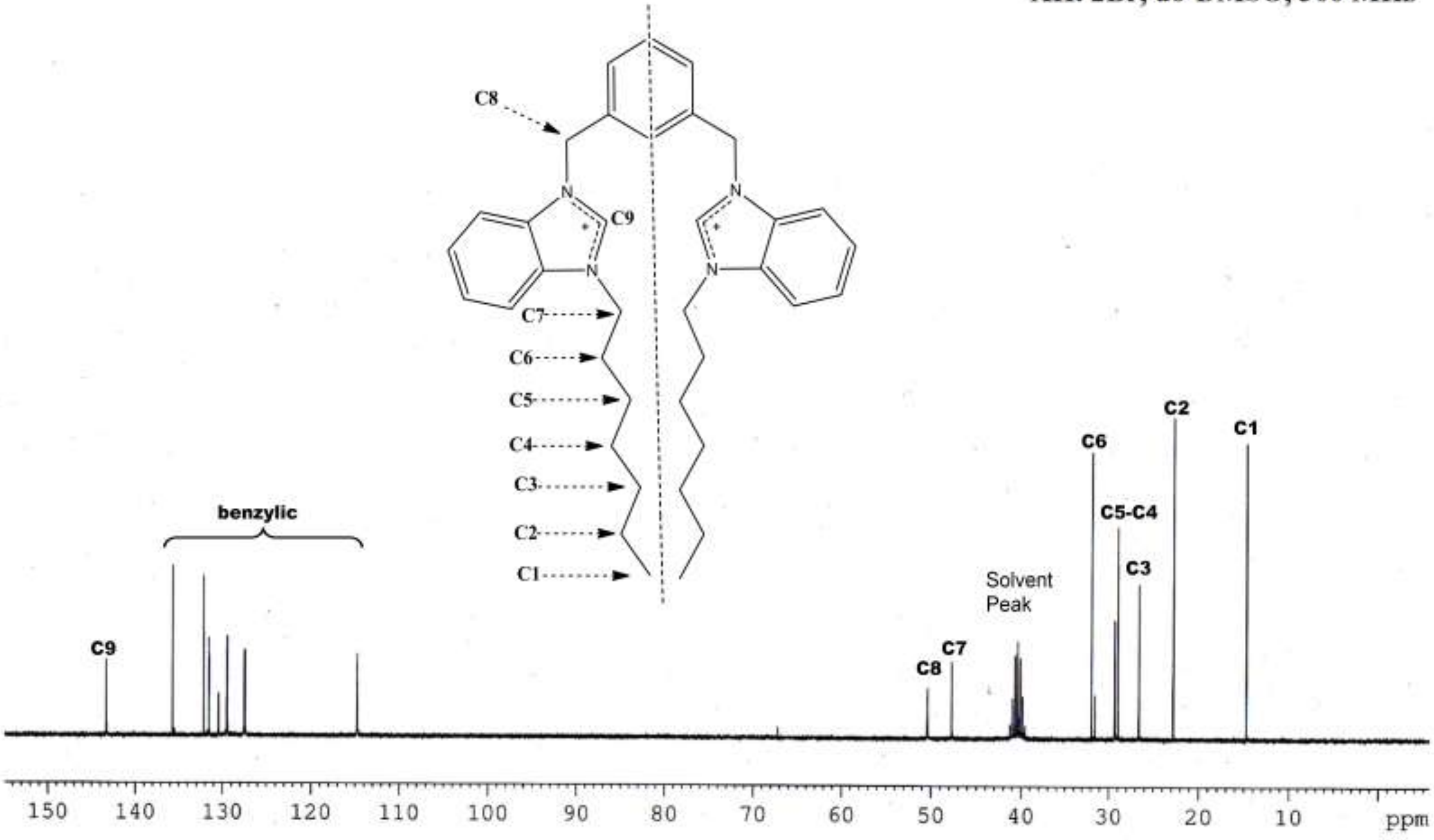

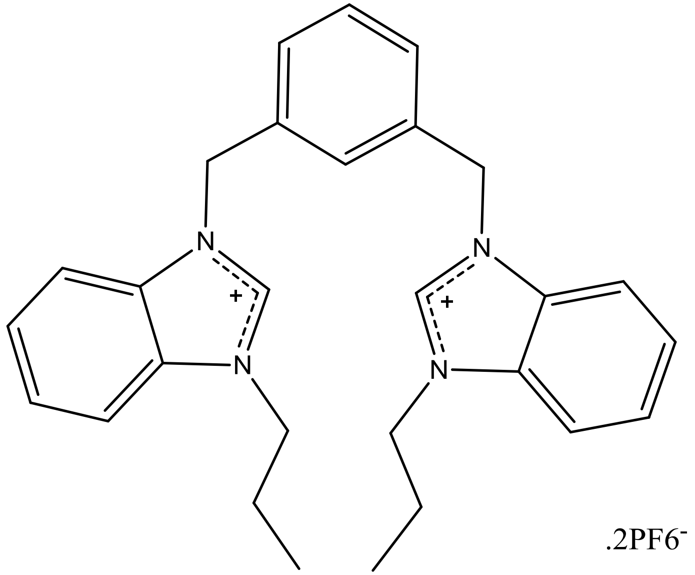

3,3'-(1,3-phenylenebis(methylene))bis(1-propyl-1H-benzimidazolium) salt (**XIII.2PF$_6$**)

1. FT-IR
2. ^{1}HNMR
3. ^{13}CNMR

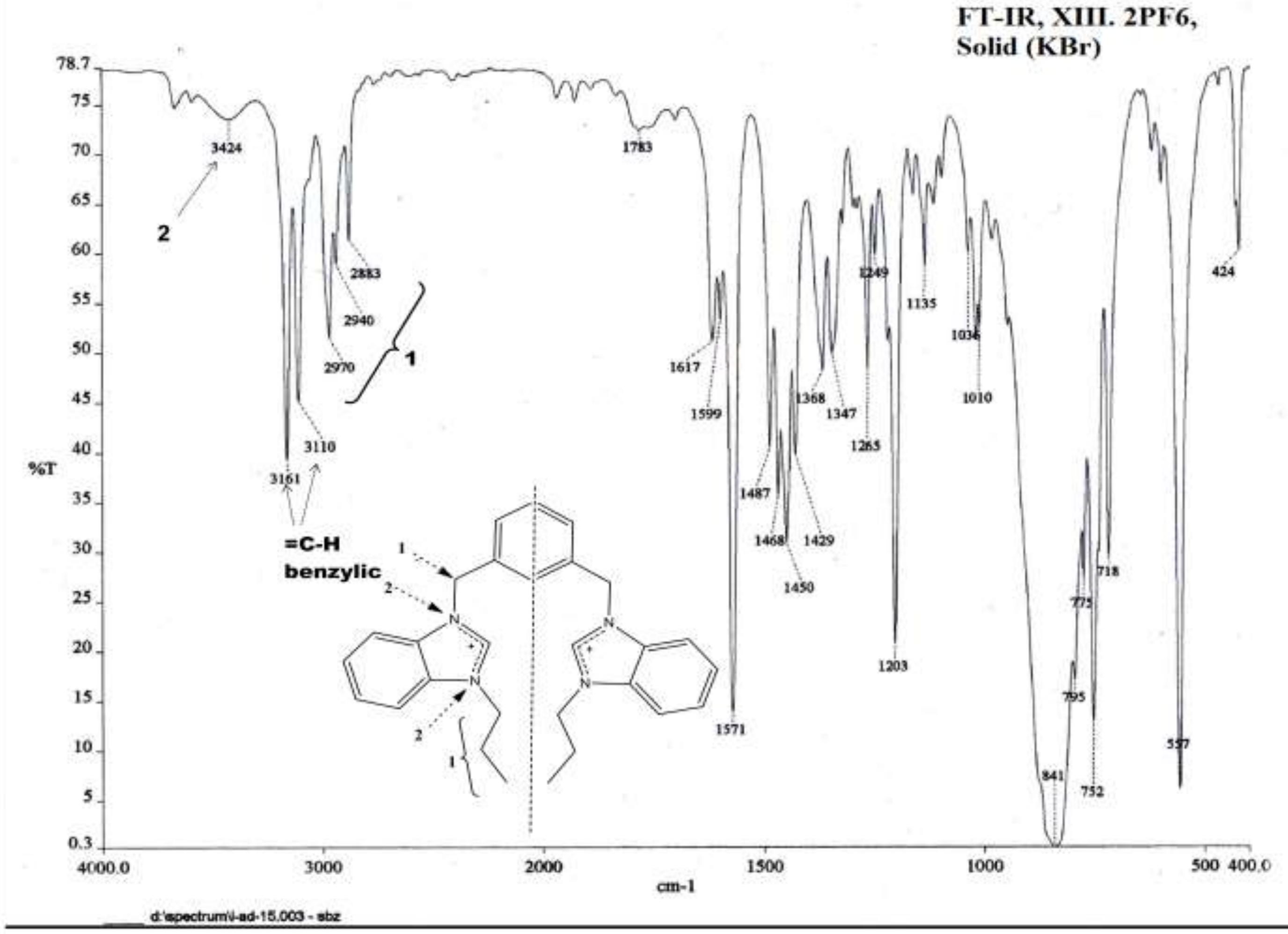
FT-IR, XIII. 2PF6,
Solid (KBr)
%T
cm-1
=C-H
benzylic
d:\spectrum\i-ad-15.003 - sbz

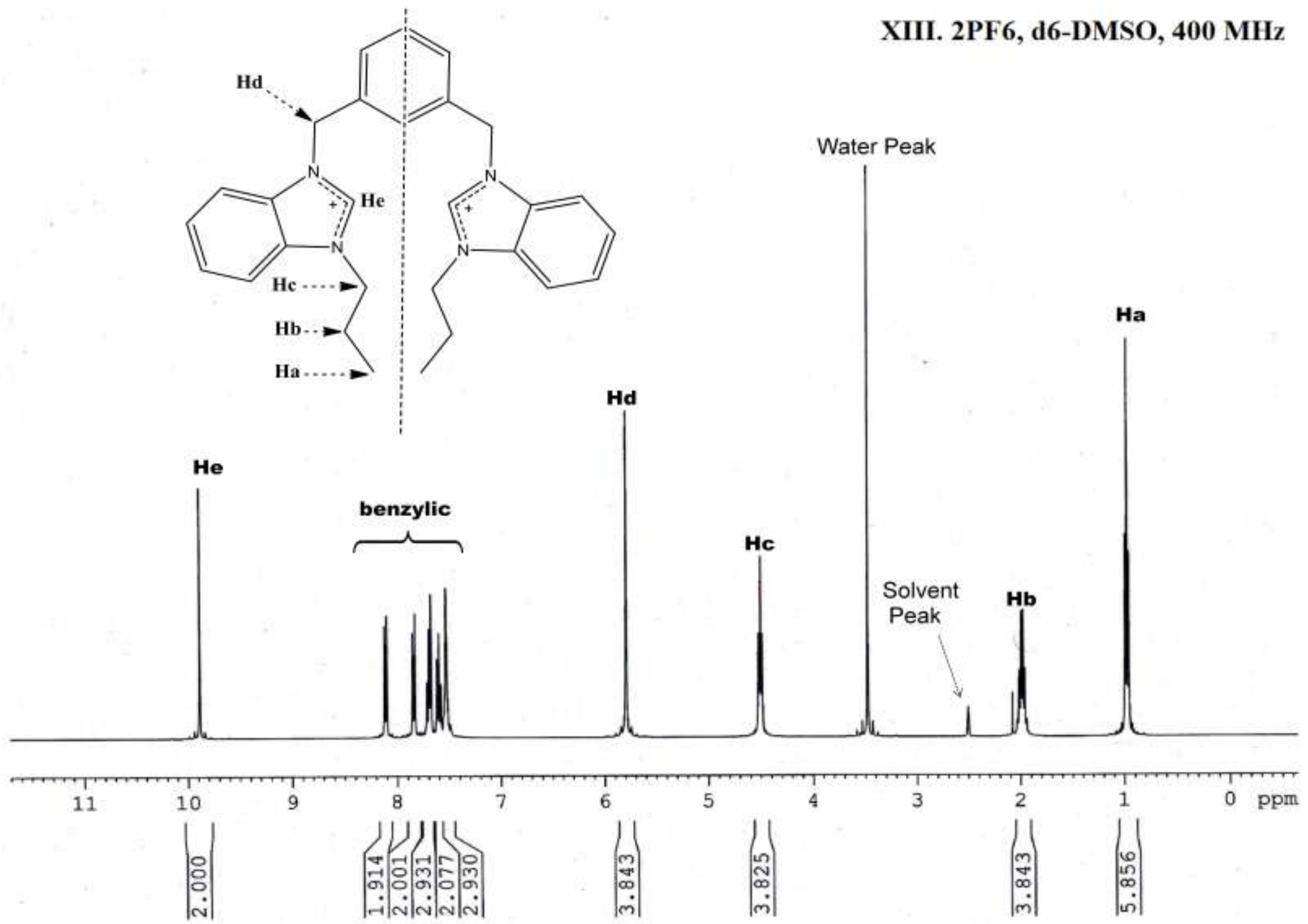
XIII. 2PF6, d6-DMSO, 400 MHz
Hd
He
Hc
Hb
Ha
Water Peak
benzylic
Solvent Peak
11
10
9
8
7
6
5
4
3
2
1
0 ppm
2.000
1.914
2.001
2.931
2.077
2.930
3.843
3.825
3.843
5.856

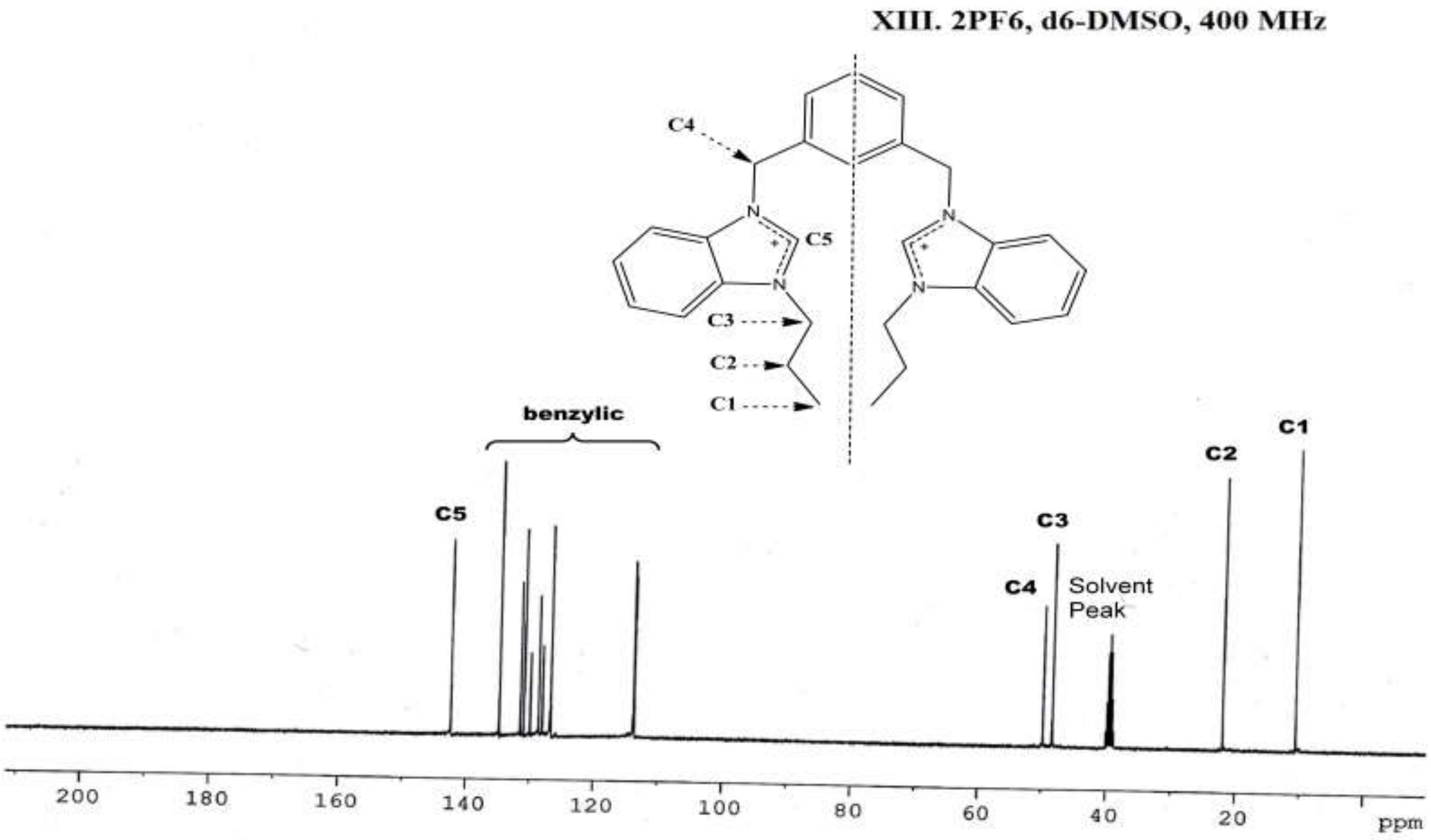
XIII. 2PF6, d6-DMSO, 400 MHz
C4
C5
C3
C2
C1
benzylic
C5
C3
C4
Solvent
Peak
C2
C1
200
180
160
140
120
100
80
60
40
20
ppm

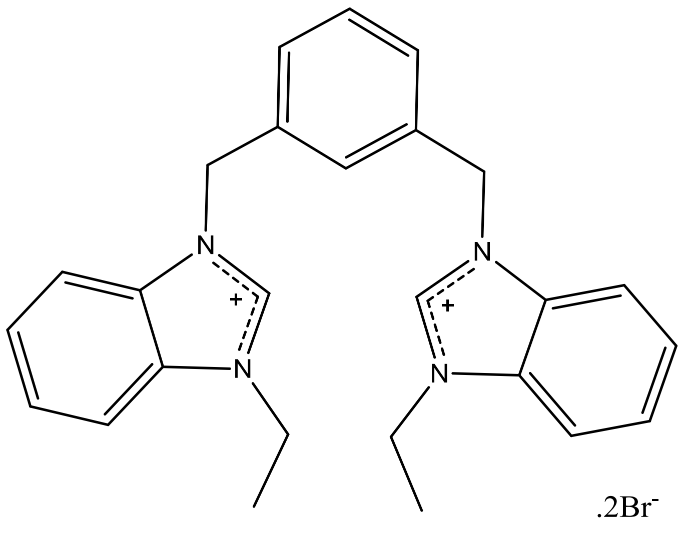

3,3'-(1,3-phenylenebis(methylene))bis(1-ethyl-1H-benzimidazolium) salt (**XIV.2Br**)

1. FT-IR
2. ^{1}HNMR
3. ^{13}CNMR

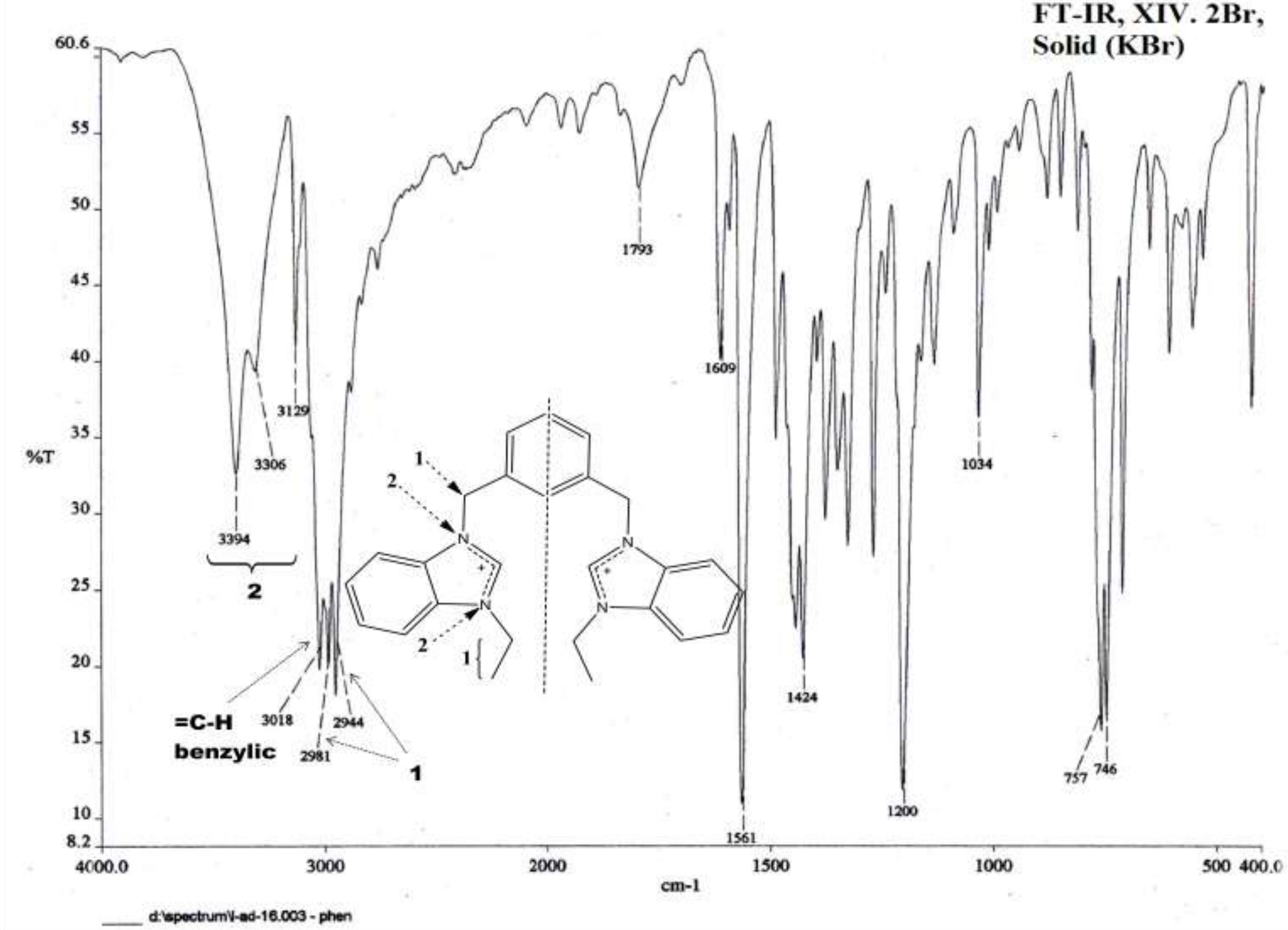
FT-IR, XIV. 2Br,
Solid (KBr)
%T
cm-1
60.6
55
50
45
40
35
30
25
20
15
10
8.2
4000.0
3000
2000
1500
1000
500 400.0
3394
3306
3129
2
3018
2981
2944
=C-H
benzylic
1
1793
1609
1561
1424
1200
1034
757
746
d:\spectrum\i-ad-16.003 - phen

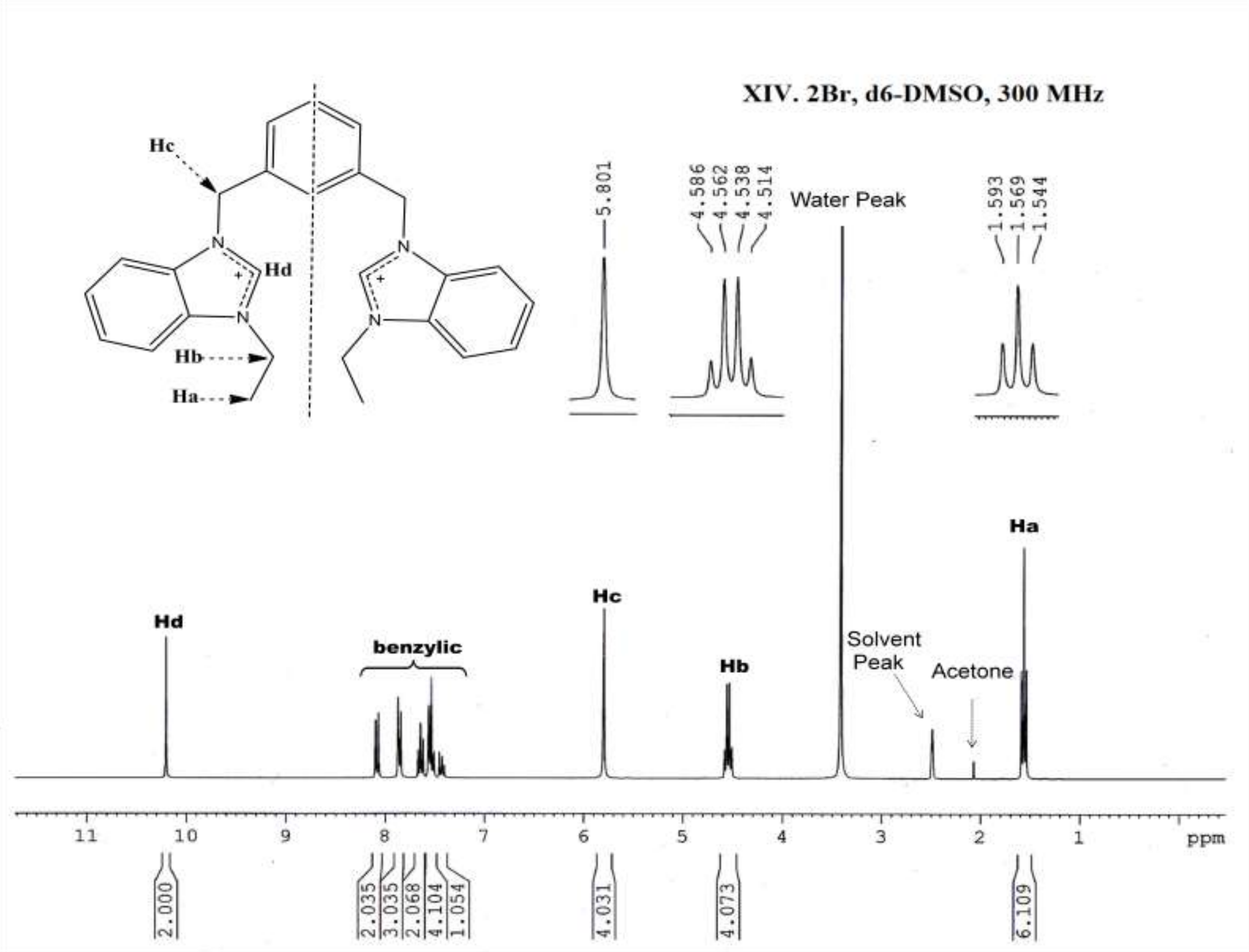
XIV. 2Br, d6-DMSO, 300 MHz
Hc
Hd
Hb
Ha
5.801
4.586
4.562
4.538
4.514
Water Peak
1.593
1.569
1.544
Ha
Hc
Hd
benzylic
Hb
Solvent Peak
Acetone
11
10
9
8
7
6
5
4
3
2
1
ppm
2.000
2.035
3.035
2.068
4.104
1.054
4.031
4.073
6.109

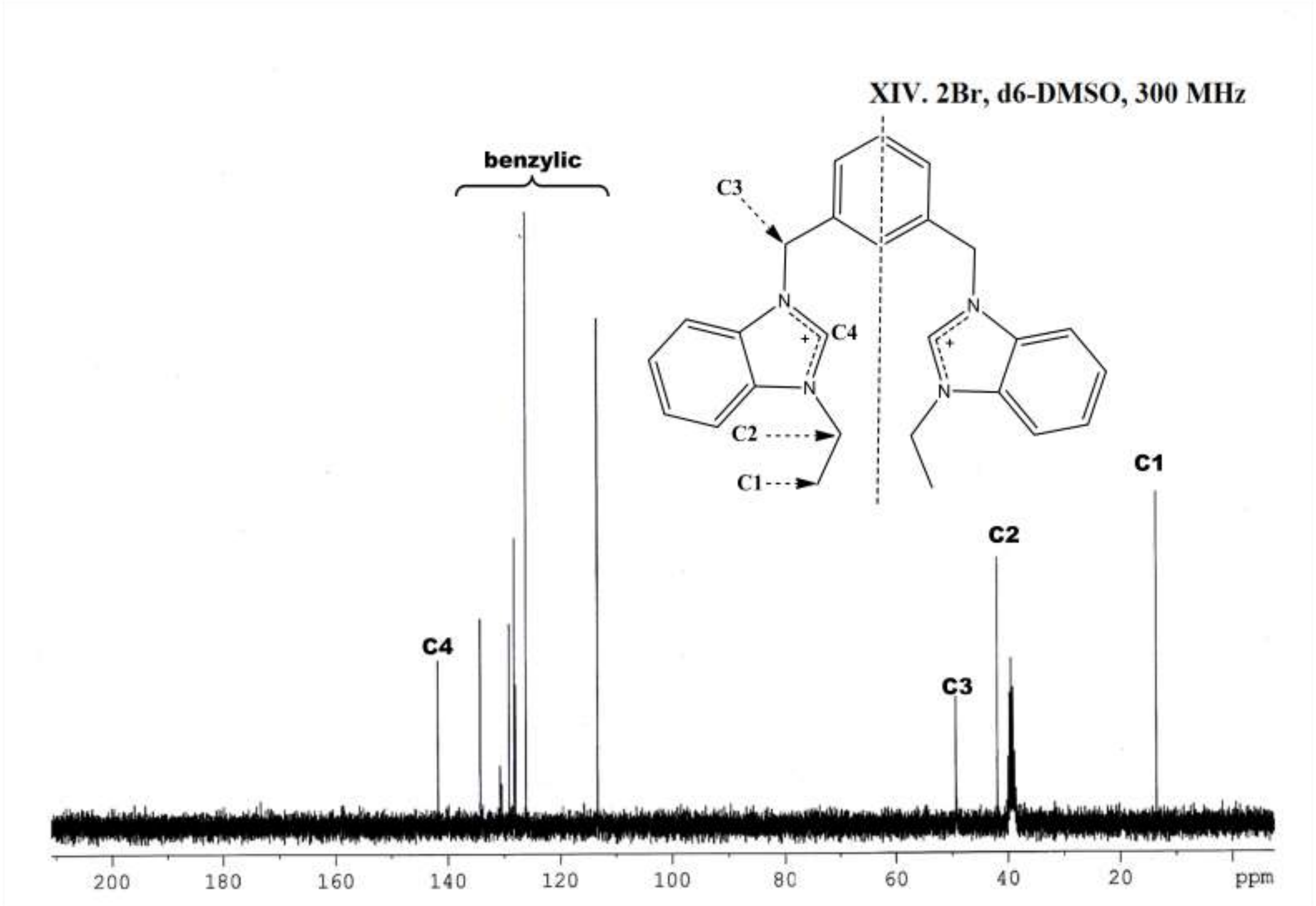
XIV. 2Br, d6-DMSO, 300 MHz
benzylic
C3
C4
C2
C1
N
+
ppm
200
180
160
140
120
100
80
60
40
20

Note:

All the spectrums were either scanned or directly saved as electronic copies on MS word from NMR PC and labelled by using MS Power Point, MS Word and ChemBioDraw 12.0.
