# Supplementary material for: Design, synthesis and structural studies of meta-xylyl linked bis-benzimidazolium salts: potential anticancer agents against ‘human colon cancer’
Source: Chem Cent J. 2012 Jul 18;6:68. doi: 10.1186/1752-153X-6-68 (PMC3542276; doi:10.1186/1752-153X-6-68)
Supplement: Additional file 2 — Table S1. Some characteristic signals of bis-benzimidazolium salts in NMR Spectroscopy. [file 1752-153X-6-68-S2.docx]

# Tables

## Table 1 - Some characteristic signals of *bis*-benzimidazolium salts in NMR Spectroscopy.

| ^1^HNMR (δ ppm) | | | | |  |
| --- | --- | --- | --- | --- | --- |
| **CH_2_-R** | **N-CH_2_-R** | **N-CH_2_-Ar** | | **NCHN** |  |
| Propyl | 4.50 | 5.79 | | 9.88 |  |
| i-Propyl | 5.05 | 5.75 | | 10.03 |  |
| Butyl | 4.50 | 5.75 | | 9.83 |  |
| Pent-2-yl | 4.96 | 5.40 | | 10.16 |  |
| Benzyl | 5.74 | 5.78 | | 10.18 |  |
| Heptyl | 4.54 | 5.82 | | 10.27 |  |
| ^13^CNMR (δ ppm) | | | | |  |
| **C-R** | **N-C-R** | **N-C-Ar** | **NCN** | |  |
| Propyl | 48.38 | 49.83 | 142.41 | |  |
| i-Propyl | 50.65 | 51.70 | 141.80 | |  |
| Butyl | 47.51 | 50.83 | 143.20 | |  |
| Pent-2-yl | 50.70 | 55.58 | 143.86 | |  |
| Benzyl | 51.11 | 51.34 | 142.30 | |  |
| Heptyl | 49.85 | 54.37 | 143.08 | |  |
